# The Effect of Methodological Considerations on the Construction of Gene-Based Plant Pan-genomes

**DOI:** 10.1093/gbe/evad121

**Published:** 2023-07-04

**Authors:** Lior Glick, Itay Mayrose

**Affiliations:** Department of Life Sciences, School of Plant Sciences and Food Security, Tel-Aviv University, Tel Aviv, Israel; Department of Life Sciences, School of Plant Sciences and Food Security, Tel-Aviv University, Tel Aviv, Israel

**Keywords:** pan-genome, assembly, annotation, gene content, presence–absence variation

## Abstract

Pan-genomics is an emerging approach for studying the genetic diversity within plant populations. In contrast to common resequencing studies that compare whole genome sequencing data with a single reference genome, the construction of a pan-genome (PG) involves the direct comparison of multiple genomes to one another, thereby enabling the detection of genomic sequences and genes not present in the reference, as well as the analysis of gene content diversity. Although multiple studies describing PGs of various plant species have been published in recent years, a better understanding regarding the effect of the computational procedures used for PG construction could guide researchers in making more informed methodological decisions. Here, we examine the effect of several key methodological factors on the obtained gene pool and on gene presence–absence detections by constructing and comparing multiple PGs of *Arabidopsis thaliana* and cultivated soybean, as well as conducting a meta-analysis on published PGs. These factors include the construction method, the sequencing depth, and the extent of input data used for gene annotation. We observe substantial differences between PGs constructed using three common procedures (de novo assembly and annotation, map-to-pan, and iterative assembly) and that results are dependent on the extent of the input data. Specifically, we report low agreement between the gene content inferred using different procedures and input data. Our results should increase the awareness of the community to the consequences of methodological decisions made during the process of PG construction and emphasize the need for further investigation of commonly applied methodologies.

**Significance**

Pan-genomics is an emerging approach for studying plant intraspecific genomic diversity by comparing multiple genomes rather than relying on a single reference. Despite the common use of pan-genomics, the methodology involved in their construction is underexplored and still poorly understood. In this study, we assess the effect of several key methodological factors on pan-genome (PG) construction and report substantial impact of the applied procedure and input data. We observe considerable differences in gene content inferences between PGs constructed from the same data using different methods and vice-versa. These findings highlight the importance of making informed decisions during the PG construction process and demonstrate the need for extensive investigation of widely used computational methodologies.

## Introduction

The continuous improvement and cost reduction of whole genome sequencing (WGS) leads to a rapid increase in the number of available plant genomes. These data provide new opportunities to study genetic diversity within and across plant species. It is now possible to examine the genetic variation present in populations consisting of hundreds or even thousands of accessions (i.e., varieties, individuals, ecotypes, or breeding lines) of the same plant species. Such comprehensive data sets may be utilized to assist crop breeding and improvement efforts (Govindaraj et al. 2015; Thudi et al. 2021) and for studying evolutionary processes at a finer scale than ever before (Wang et al. 2020; Xia et al. 2020). Traditionally, diversity analyses were conducted by means of “genome resequencing”—a procedure in which sequencing data from multiple plant accessions are compared with a single reference genome, thus allowing the detection of genetic variation at genomic regions present in the reference genome. In recent years, a new approach inspired by comparative studies of microorganisms has emerged, which applies the concept of a species' pan-genome (PG) (Gordon et al. 2017; Gao et al. 2019; Hübner et al. 2019).

The PG is defined as the nonredundant pool of genetic material present in the population of a given species (or a broader taxonomic group) (Lei et al. 2021). In practice, PG studies typically examine a sample comprising dozens, hundreds, or even thousands of accessions, taken to represent the majority of the intraspecific diversity. Rather than comparing short sequence segments with the reference genome, genomes of multiple accessions are assembled and directly compared with one another. This has several advantages over the single-reference approach, with the most prominent one being the ability to detect and analyze novel genomic sequences that are not present in the reference genome. These nonreference sequences may potentially contain functional elements, with protein-coding genes being of particular interest and thus the focus of most PG studies conducted to date (Bayer et al. 2020). The overall pool of genes found within the examined accessions is therefore divided into the reference and the nonreference (or novel) gene sets. Additionally, PGs provide information regarding gene presence–absence variation (PAV) across accessions, which helps to correlate the presence and absence of specific genes or groups of genes with genetic, morphological, or physiological attributes (Ou et al. 2018).

When focusing on protein-coding sequences (“gene-based” PGs), PGs are composed of a set of pan-genes, each representing an orthologous set of genes present in one or more of the studied accessions. Pan-genes vary in terms of the number of accessions in which they are present. Pan-genes found in all examined accessions are termed core genes, whereas those present in some accessions only are referred to as shell or dispensable genes. Genes that are found in a single accession are called singletons. The relative ratios of core, shell, and singleton pan-genes, sometimes referred to as the PG composition, were previously hypothesized to be characteristic of particular plant species, potentially reflecting their evolution, ecology, and life history (Bayer et al. 2020).

In recent years, a different approach for pan-genomics has been developed, in which the term PG is used to represent a graph data structure, constructed based on inferred structural variants (SVs). These SVs are inferred from comparisons of whole genome sequences or long-read sequencing (Hübner 2022). Multiple tools have been devised to facilitate the construction of graph-based PGs (Garrison et al. 2018; Li et al. 2020; Guarracino et al. 2022; Hickey et al. 2022). Utilizing such graphs, instead of a single-reference genome, for mapping short reads enhances genotyping capabilities. Although graph-based PGs have been effective in analyzing large SVs (Hickey et al. 2020; Ebler et al. 2022), there is currently a lack of standardized protocols for utilizing them in the inference of gene content variation. Thus, many studies construct both graph-based and gene-based PGs and analyze them independently (Hufford et al. 2021; Tao et al. 2021; Li et al. 2022). Furthermore, graph-based analyses heavily depend on the availability of high-quality (HQ) genome assemblies (Jayakodi et al. 2020; Liu et al. 2020), which can be challenging to obtain for PGs containing a large number of accessions. As a result, such efforts are not commonly pursued. In this study, we focus on methodological considerations for the construction of gene-based PGs.

**Fig. 1.**
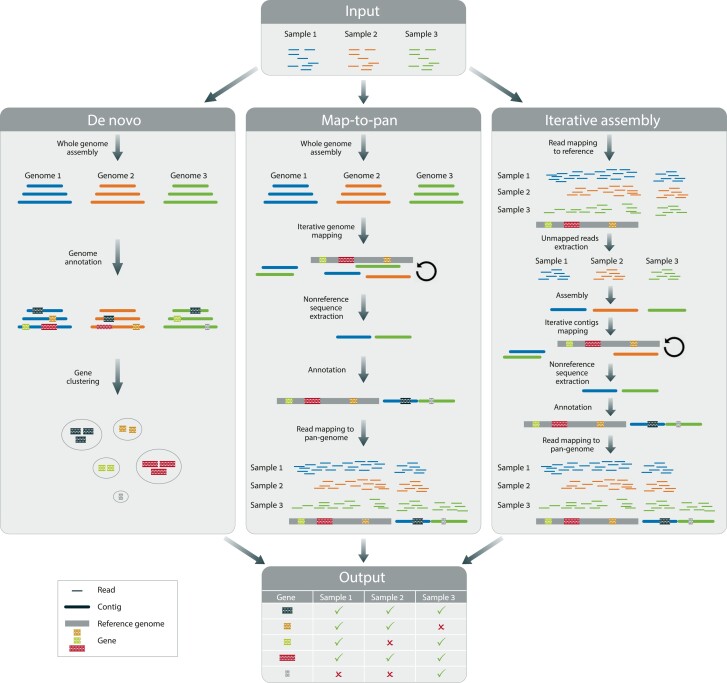
The general workflows of the DN, MTP, and IA approaches for PG construction. All approaches start from raw sequencing reads as input. The DN approach (left) begins with a whole genome assembly procedure applied to the sequencing reads of each accession, resulting in longer genomic sequences (contigs). Next, whole genome annotations of each assembled genome are performed. Gene models are detected and then clustered based on sequence similarity, with each cluster representing a pan-gene. Gene presence–absence per accession is determined based on the existence of representative genes within clusters. The MTP approach (middle) also begins with whole genome assembly but proceeds with an iterative mapping procedure to detect novel (nonreference) genomic sequences. Gene annotation is only performed on these regions, and nonreference gene models are predicted. Next, gene presence–absence is determined based on mapping of sequencing reads and analysis of gene sequence coverage in each accession. In the IA approach (right), reads are first mapped to the reference genome, and only unmapped reads are assembled into contigs which are then subject to the same steps as described for the MTP approach. All approaches result in a matrix indicating the presence or absence of each pan-gene across the accessions included in the PG.

Notably, the construction of a plant gene-based PG is a laborious, time-consuming, and often an expensive task, requiring considerable amounts of sequencing data as well as computational resources. Several gene-based PG construction methods have been devised, most of which can be classified as variants of three general methodologies: the de novo (DN) assembly and annotation approach, the map-to-pan (MTP) approach, and the iterative assembly (IA) approach. The general workflows of these approaches are depicted in figure 1 (also see Golicz et al. (2016) and Hu et al. (2020) for a comprehensive review). Briefly, in the DN approach, the genome of each accession is first assembled, and then, the entire genome sequence is automatically annotated to predict gene models (i.e., predicting protein-coding gene loci and exon–intron structures). The obtained gene sequences are then subject to homology clustering, allowing the definition of pan-genes as well as the identification of nonreference genes and PAVs. The MTP approach also begins with whole genome assembly but avoids the intense computations required for whole genome annotation by applying the annotation procedure only to novel (i.e., nonreference) genomic sequences. These nonreference sequences are detected using an iterative mapping procedure in which assembled contigs showing low homology to the reference are added to the PG and annotated (i.e., scanned for the presence of nonreference genes). Gene presence–absence is determined by mapping the sequencing reads of each accession to the set of genomic sequences contained in the PG: a gene is determined as present in a given accession if it is sufficiently covered by reads from that accession. The IA procedure is essentially similar to MTP, but here, computational requirements are further reduced by avoiding both whole genome assembly and whole genome annotation. Accordingly, reads of each accession are first mapped to the reference genome and only unmapped reads are assembled. Nonredundant assembled contigs from multiple accessions are added to the PG and annotated. Similar to MTP, gene presence–absence is determined based on mapping the raw sequencing reads to the PG. A few studies have applied a variant of the IA approach, inspired by metagenomic methodologies (Yao et al. 2015; Zhao et al. 2020). The unmapped reads from all accessions are pooled together and assembled into contigs, which are then annotated. This strategy is useful in cases where the sequencing depth of individual accessions is particularly low, such that the assembly of each individual accession is challenging. However, it may result in chimeric contigs that are not present in any of the accessions (reviewed in Lei et al. 2021) and is thus less commonly applied.

In all construction approaches, gene annotation may be based on RNA-seq data produced for a subset of the analyzed accessions (Gao et al. 2019), protein homology to other plant species (Hübner et al. 2019), ab initio prediction (Wang et al. 2018), or a combination of these. This procedure allows the identification of gene models that do not exist in the reference genome (i.e., nonreference genes). Another possible annotation approach, sometimes termed “gene projection", has been applied for very large and complex genomes such as those of cereals (Haberer et al. 2020; Jayakodi et al. 2020; Walkowiak et al. 2020). In this approach, HQ gene models are produced for a small number of accessions and mapped to the rest of the analyzed collection, thus avoiding the need to fully annotate multiple genomes. However, the discovery of novel nonreference genes is limited to the small number of accessions for which full genome annotation is applied, thus potentially overlooking many novel genes.

Several past publications have discussed the expected differences between the various PG construction approaches and the effects of other relevant methodological factors. Golicz et al. (2016) discussed several methodological and technical factors that may affect the construction of a PG. These include the quality of genome assemblies and annotations, the gene orthology detection method applied, and of course the set of selected accessions. Other reviews have discussed the expected differences between the various construction approaches, potential advantages and shortcomings of the methods, and scenarios in which they should be applied (Bayer et al. 2020; Golicz et al. 2020; Hu et al. 2020). For instance, it was hypothesized that PGs constructed using the MTP or IA approach could overlook variation within genomic regions that display high homology to the reference sequence. Thus, for example, a frameshift in the coding sequence caused by a single-nucleotide insertion could severely disrupt the gene product, leading to a pseudogenization event. In such cases, the gene will likely be inferred as absent by the DN approach but present by the MTP or IA approaches because the genomic region would still be well covered by mapped reads. It was also suggested that arbitrary choices of parameter values used within each construction approach may affect the analysis and lead to different inferences regarding the composition and content of a PG (Lei et al. 2021). For example, gene orthology clusters produced by the DN approach may be influenced by methodological factors (Natsidis et al. 2021). These past studies provide indications that the construction approach, as well as many other methodological factors, could affect the resulting PG. Nevertheless, there is no study, to date, that directly evaluated, based on the same data, the effect and extent of such factors. The consequences of various decisions made throughout a PG analysis therefore remain unclear.

PG projects also differ considerably in the input data used for their construction, from the amount (depth) and type of sequencing data used for genome assembly to the quality and richness of the data used in genome annotation. The amount and quality of input data often reflects the purpose and the budget of a project, specifically the number of analyzed accessions. Still, better understanding of the effects of such factors may assist researchers in making more informed decisions and better use of their budgets.

In this study, we explore the effect of different methodological considerations on the resulting PG. Specifically, we examine the effects of the construction approach (DN, MTP, or IA), the depth of sequencing, the quality of assembly, and the quality of annotation evidence on the two main aspects of the resulting PG: nonreference gene detection and gene presence–absence inference. This was achieved by constructing multiple PGs using different procedures and empirical data sets and comparing them with one another. In addition, we explore the effects of several technical parameters specific to each approach. Our analysis reveals that certain factors affect the constructed PG to different extents and sometimes in ways that would be difficult to predict.

## Results

In the following sections, we investigate the impact of different methodological factors on the construction of gene-based PGs. We employed a range of procedures and data sets to construct 20 *Arabidopsis thaliana* and 7 cultivated soybean (*Glycine max*) PGs, as detailed in supplementary table S1, Supplementary Material online. By comparing these PGs with one another, we were able to evaluate the effect of the analyzed methodological factors on various aspects of the resulting PGs.

### The Effect of the PG Construction Approach

We first examined the effect of the construction approach on the resulting PG. To this end, we constructed three PGs from the same input data using the DN, MTP, and IA procedures (fig. 1). All three PGs encompassed the same eight *A. thaliana* ecotypes (supplementary table S2, Supplementary Material online), including the reference accession Col-0 and seven additional ecotypes, for which sufficient raw Illumina sequencing data are available. In this first comparison, the sequencing reads were subsampled to a depth of 50×.

**Fig. 2.**
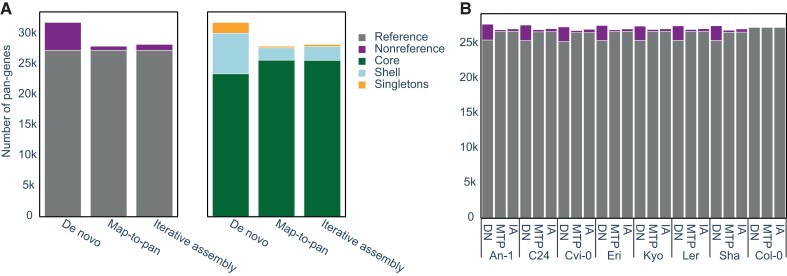
Comparison of *A. thaliana* PGs constructed using the DN, MTP, and IA approaches. The same input data were used for constructing three PGs containing eight *A. thaliana* ecotypes (accessions), using the DN, MTP, and IA approaches. (*A*) The overall PG sizes and compositions—number of reference and nonreference pan-genes, as well as core, shell, and singletons. (*B*) Number of reference and nonreference pan-genes detected by the three approaches in each ecotype.

Considerable differences were observed between the constructed PGs (fig. 2*A* and table 1). The total number of pan-genes in the DN PG was 14% and 13% larger than those obtained using the MTP and IA approaches, respectively. Because the number of reference genes in all three PGs is identical, this difference stems solely from a substantial increase in the number of nonreference pan-genes in the DN PG (DN, 4,565; MTP, 663; and IA, 959). Moreover, the composition of the PGs differed markedly, with the proportion of shell genes approximately three times higher in the DN PG compared with that of the MTP and IA approaches. In contrast, the fraction of core genes was considerably higher in the MTP PG (91.8%) and the IA PG (90.7%) compared with the DN PG (73.5%). Notably, although the total number of genes identified as present in each ecotype was consistently lower in the MTP and IA PGs compared with the DN PG (average difference of 606 ± 111 and 469 ± 110 genes for MTP and IA, respectively), the number of present reference genes was consistently higher (average difference of 1,253 ± 43 and 1,261 ± 32 genes for MTP and IA, respectively; fig. 2*B* and supplementary table S3, Supplementary Material online). Together, these results indicate that (1) the DN approach detects more novel, nonreference genes and (2) the MTP and IA approaches tend to identify more genes as present in all accessions, that is, belonging to the core genome. These findings are in line with previous literature and may be expected because the DN approach captures variation in whole genome sequences, whereas the MTP and IA approaches only examine sequences with low homology to the reference. Importantly, this does not imply that one approach performs better than others but rather that each construction procedure leads to a considerably different PG.

**Table 1:** Statistics for *A. thaliana* and Soybean PGs Constructed Using the DN, MTP, and IA Approaches

	*A. thaliana*			Soybean		
	De Novo	Map-to-Pan	Iterative Assembly	De Novo	Map-to-pan	Iterative Assembly
Total pan-genes	31,860	27,958	28,254	70,898	52,058	52,287
Reference pan-genes	27,295 (85.7%)	27,295 (97.6%)	27,295 (96.6%)	51,769 (73.02%)	51,769 (99.44%)	51,769 (99%)
Nonreference pan-genes	4,565 (14.3%)	663 (2.4%)	959 (3.4%)	19,129 (26.98%)	289 (0.56%)	518 (1%)
Core pan-genes	23,426 (73.5%)	25,657 (91.8%)	25,622 (90.7%)	38,618 (54.47%)	51,027 (98.02%)	51,086 (97.7%)
Shell pan-genes	6,651 (20.9%)	2,041 (7.3%)	2,283 (8.1%)	24,197 (34.13%)	953 (1.83%)	1,106 (2.1%)
Singletons	1,783 (5.6%)	260 (0.9%)	345 (1.2%)	8,083 (11.4%)	77 (0.15%)	95 (0.18%)

One of the central aims of constructing PGs is the discovery of nonreference genes, because these genes cannot be detected using a single reference genome. We thus focused the next comparison on the sets of nonreference genes. Due to the general similarity between the MTP and IA procedures, we focused on the MTP and DN nonreference gene pools (see supplementary note 1, Supplementary Material online, for a comparison between the MTP and IA approaches). We assessed the reliability of nonreference gene predictions by applying a series of analyses to each nonreference gene (table 2). First, protein sequences were searched against the reference *A. thaliana* proteome. This search revealed that 2.3% (104) of the DN nonreference proteins and 5.7% (38) of the MTP nonreference proteins were highly similar to reference proteins (sequence identity ≥ 95% and 0.8 < length ratio < 1.2). An additional 7.5% (341) and 3.5% (23) of the DN and MTP nonreference proteins, respectively, were found to be truncated versions of reference proteins (sequence identity ≥ 95% and length ratio ≤ 0.8). In both cases, the identification of these genes as nonreference is likely due to the existence of a very close paralog or an artifact of the PG construction and annotation procedure. Next, we searched for additional functional support for the existence of nonreference genes. Potential homologs of the nonreference proteins were identified by searching through a database of plant proteins (Ensembl Plants (Cunningham et al. 2022), excluding *A. thaliana*). Homologs were found (sequence identity > 50%) for 14% (639) and 32.9% (218) of the DN and MTP proteins, respectively. Additionally, using an extensive RNA-seq data set, evidence of nonreference gene expression was found (reads per kilobase of transcript, per million mapped reads [RPKM] > 1) for 12.9% (588) of the DN nonreference genes and 27.3% (181) of the MTP nonreference genes. We defined HQ candidates as nonreference genes that do not highly resemble reference proteins but do show homology to other known plant proteins or evidence of being transcribed. We found that 18.7% (856) and 39.4% (261) of the DN and MTP nonreference genes are HQ candidates. These results suggest that the higher number of gene models predicted by the DN approach allows it to detect more genuine novel genes, compared with the MTP approach. Note, however, that other definitions for HQ candidates are possible and that results may be dependent on the quality of the sequence data available in the target databases.

**Table 2:** Statistics on the Reliability of Nonreference pan-genes in *A. thaliana* PGs Constructed Using the DN and MTP Approaches

	De Novo	Map-to-Pan
Total nonreference pan-genes	4,565	663
High similarity to reference	104	38
Truncated reference	341	23
Has plant homolog	639	218
Expressed	588	181
High-quality candidates	856	261

Out of the 4,565 and 663 nonreference pan-genes found in the DN and MTP PGs, respectively, 89 could be reliably matched between approaches based on protein sequence similarity. Thus, the great majority of nonreference genes were only detected by one of the pipelines but not the other. Hereafter, pan-genes that are only present in the nonreference pool of the DN pipeline are termed DN+|MTP−, whereas pan-genes only detected by the MTP pipeline are termed DN−|MTP+. By applying a series of transcript and protein sequence mapping analyses, we identified multiple methodological causes for the existence of DN+|MTP− and DN−|MTP+ pan-genes. For instance, using the MTP approach, it is more challenging to detect nonreference genes in regions that are highly similar to reference regions annotated as noncoding, which leads to DN+|MTP− nonreference genes (supplementary note 2, Supplementary Material online and supplementary fig. S1, Supplementary Material online). On the other hand, methodological factors related to the orthology clustering step of the DN approach may lead to DN−|MTP+ genes (supplementary note 3, Supplementary Material online and supplementary fig. S1, Supplementary Material online).

The analyses described above concentrated on the overall gene content of the compared PGs. Next, we examined discrepancies in gene presence–absence within each ecotype by comparing the gene PAV matrices of the two PGs. In this analysis, we omitted genes that could not be matched across the two PGs (i.e., DN+|MTP− and DN−|MTP+), leaving 89 matched nonreference genes as well as 27,295 reference genes. Out of these, 23,106 (84.4%) were detected as core genes in both PGs. However, because these, by definition, display no variation and are present in all ecotypes, they are of lesser interest in the context of a PG analysis. We therefore proceeded only with the 4,278 pan-genes detected as noncore by either of the PG construction approaches. A total of 29,946 gene presence–absence calls were performed across these pan-genes (4,278 pan-genes times the 7 nonreference ecotypes). Notably, we observed complete agreement in gene presence–absence assignments between the two approaches in only 13% of these pan-genes. Moreover, 39% of the presence–absence calls performed for these noncore genes were different in the two PGs. Most of these differences (88%) were classified as present in the MTP PG and absent in the DN PG, with various methodological causes identified (supplementary note 4, Supplementary Material online). These results indicate that the MTP approach tends to more readily identify annotated genes as present (but see supplementary note 5, Supplementary Material online, for the dependency of the MTP approach on the chosen parameters used for gene presence–absence detection) and further emphasize the observation that different PG construction strategies result in substantially different PGs with regard to shell and singleton genes.

### The Effect of Sequencing Depth and Assembly Quality

Regardless of the construction approach applied, it is expected that the depth of the input sequencing data will affect the quality of the obtained PG. We assessed this effect by subsampling the raw reads of each accession to produce data sets with sequencing depths 10×, 20×, 30×, and 50× as well as an additional data set that consists of the full sequence data, with average depth of 78×. Each data set was used as the input for the DN and MTP construction pipelines. Two additional PGs, termed “HQ-assembly” PGs, were constructed by applying the DN and MTP pipelines to a previously published data set of HQ, chromosome-level genome assemblies of the seven ecotypes (Jiao and Schneeberger 2020). These HQ-assembly PGs serve two purposes: first, they represent cases in which PGs are constructed from HQ genome assemblies, and second, they are used as a baseline for assessing the quality of PGs produced from the various data sets. We further note that constructing PGs based on short-read 10× data, especially using the DN approach, is usually not a realistic scenario, and it is used here merely to demonstrate the effects of low sequencing depth.

**Fig. 3.**
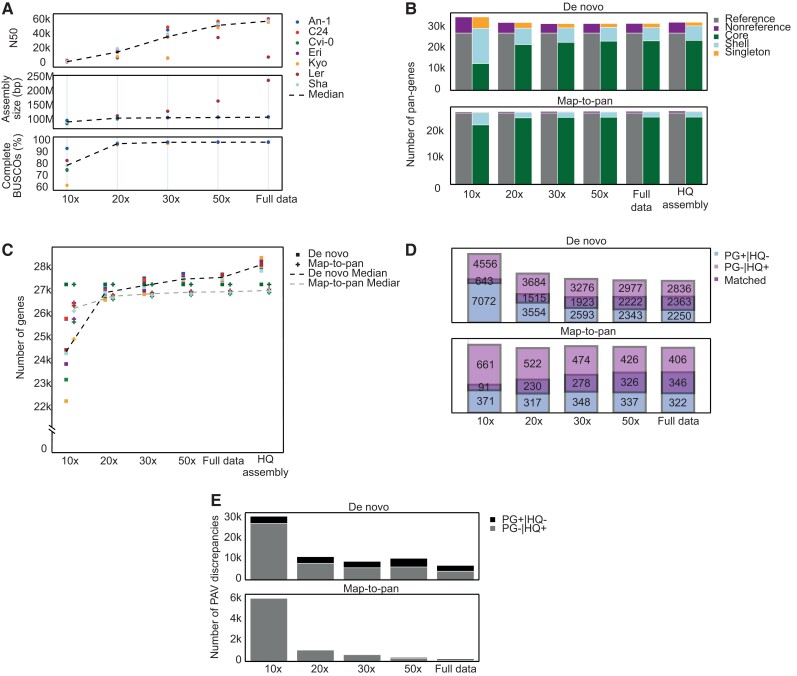
The effect of assembly quality on *A. thaliana* PG construction. PGs were constructed using the DN and MTP approaches on data sets of increasing sequencing depth. All PGs were compared with a PG constructed using HQ assemblies. (*A*) The effect of sequencing depth on three assembly quality measures: contig N50 (top), assembly size (middle), and % complete BUSCOs (bottom) across ecotypes. (*B*) The effect of assembly quality on DN (top) and MTP (bottom) PG sizes and compositions. (*C*) The effect of assembly quality on the number of genes detected in each ecotype when using the DN (squares) and MTP (crosses) approaches. Note that the *y* axis starts at 22,000 genes. (*D*) Comparisons of nonreference gene pools of each PG to the HQ PG constructed using the same approach (top, DN; bottom, MTP). Rectangular Venn diagrams show the proportions of PG+|HQ− (light blue), PG−|HQ+ (light purple), and matched (overlapping area) pan-genes in each comparison. (*E*) Comparisons of gene presence–absence calls of each PG to the HQ PG constructed using the same approach (top, DN; bottom, MTP). The numbers and types of presence–absence discrepancies were calculated while only considering reference and matched nonreference genes which are not core in both PGs.

Both the DN and MTP pipelines begin the construction process by creating whole genome assemblies of all input accessions. As expected, assembly contiguity (contig N50) and completeness (assembly size and % complete BUSCOs (Waterhouse et al. 2018)) improved as sequencing depth increased (fig. 3*A*, supplementary table S4, Supplementary Material online, and supplementary note 6, Supplementary Material online). The high BUSCO scores (% complete BUSCOs higher than 96% for all ecotypes) obtained with sequencing depth as low as 20× suggests that assemblies of the gene space with satisfying quality could be produced from WGS data commonly used for variant detection in genome resequencing studies (although this will probably not be the case for larger and more complex genomes).

The total number of detected pan-genes was mainly affected by the construction approach, rather than the sequencing depth, ranging from 31,811 to 32,494 for DN PGs and from 27,757 to 28,047 for MTP PGs (fig. 3*B* and supplementary table S5, Supplementary Material online). The exception to this was the DN 10× PG, which contained a considerably larger number of pan-genes (35,010), suggesting a high frequency of erroneously annotated genes, possibly due to gene model fragmentation over multiple contigs. Similarly, we found that PG composition was affected by assembly quality only at very low sequencing depths, and the proportions of core pan-genes reached a plateau at high sequencing depths. For instance, only 36.2% of the genes were classified as core genes in the DN 10× PG, compared with 73.5% and 73.2% in the DN 50× and HQ-assembly data sets, respectively (fig. 3*B*). This effect is also observed, although to a lesser degree, when comparing MTP results, with 81.9% and 91.7% core genes in the MTP 10× and HQ-assembly PGs, respectively. The number of genes detected as present in each ecotype increased with the sequencing depth (fig. 3*C* and supplementary table S6, Supplementary Material online). Particularly low numbers of genes were observed for the DN 10× PG (median number of present genes across ecotypes 24,419). This is despite the overall high number of pan-genes included in the gene pool of the 10× PG and in line with the lower quality of results produced with such low sequencing depth. Taken together, these results suggest that improving assemblies beyond a certain quality has a relatively modest effect on the resulting PG. Thus, even if genome assemblies of the highest quality are obtained using multiple sequencing technologies, other methodological factors still affect the results, causing considerable differences between PGs constructed using the DN and MTP approaches. It should be noted, however, that this conclusion may not hold for plant species with larger and more complex genomes (e.g., cereals), for which obtaining HQ assemblies may have a considerable advantage. Furthermore, obtaining HQ genomes may serve other purposes besides gene-based pan-genomics, such as the detection of SVs.

We next focused on the effect of assembly quality on the ability to detect nonreference pan-genes (fig. 3*B* and supplementary table S7, Supplementary Material online). Using the MTP approach, the number of predicted nonreference genes was not considerably affected by assembly quality. In contrast, in the DN PGs, the number of nonreference pan-genes slightly decreased as assembly quality improved. This could be explained by a high frequency of erroneously detected genes resulting from fragmented or misassembled genomes in low-quality assemblies. To further evaluate the effect of assembly quality on nonreference gene detection, we compared the nonreference gene pool of each PG with the PG constructed with the same approach using the HQ assemblies. These HQ PGs serve as a proxy for the set of nonreference genes detected when assembly quality is not a limiting factor. Each pan-gene was therefore classified as “matched” (found in both the tested and the HQ-assembly PGs), “PG−|HQ+” (found only in the HQ-assembly PG), or “PG+|HQ-” (found only in the tested PG). As shown in figure 3*D*, the proportion of matched nonreference pan-genes gradually increased as assembly quality improved in both the DN and MTP data sets, whereas the proportions of PG+|HQ− and PG−|HQ+ generally decreased. However, in all comparisons, there were relatively small overlaps in the nonreference gene content produced by PGs of different qualities, even when using the full sequencing data set (ca. 78×).

We proceeded by investigating the effect of sequencing depth on the accuracy of gene presence–absence detection per ecotype. We compared the gene PAV matrix of each PG with that of the corresponding HQ-assembly PG while ignoring unmatched nonreference pan-genes, as well as pan-genes classified as core in both PGs. In each comparison, we determined the percentage of agreement in gene presence–absence calls as well as the proportions of the two types of discrepancies: PG−|HQ+ and PG+|HQ−. Although the percentage of agreement in presence–absence calls increased in accordance with sequencing depth for both construction methods, it was consistently higher in MTP PGs compared with DN PGs constructed with the same data (fig. 3*E* and supplementary table S8, Supplementary Material online). For example, 20.2% of all gene presence–absence calls were in disagreement between the DN 50× and the DN HQ assemblies PGs, compared with only 2.4% between the corresponding MTP PGs. For both DN and MTP data sets, the increased agreement of presence–absence calls in data sets with higher sequencing depth is mainly explained by a decrease in the number of PG−|HQ+ discrepancies, whereas the number of PG+|HQ− discrepancies remained similar across data sets. These results indicate that the accuracy of gene presence–absence calling can be enhanced by deeper sequencing in both construction pipelines, although for the MTP approach, increasing the depth beyond 50× has a negligible effect.

Finally, we determined the effect of using long-read sequencing data as input for the DN and MTP procedures. When using long-read data, a common practice is to use a combination of short and long reads to produce a “hybrid” assembly. We thus produced a sequencing data set with a total depth of 50×, comprising 25× short reads and 25× long reads. On average, hybrid assemblies were more contiguous than those obtained using 50× short reads (supplementary table S4, Supplementary Material online). However, PGs constructed using both approaches were comparable with those obtained using the 50× short-read data set (supplementary tables S5–S8, Supplementary Material online). For example, the total number of pan-genes was 2% larger in the hybrid PG using the DN approach (32,527 vs. 31,860) and 0.2% smaller when using the MTP approach (27,891 vs. 27,958). Likewise, the proportions of core, shell, and singleton pan-genes differed by no more than 2.4% between the hybrid and the short-read 50× PGs, using either construction approach.

### The Effect of Annotation Evidence

Genome annotation is a crucial step in the process of PG construction because it facilitates the detection of novel genes and highly affects the composition of the constructed PG. However, automatic genome annotation is often a nontrivial task requiring various inputs and involving multiple computational steps. The quality of the result is determined by many factors, including the specific tools used and the parameters set for each of them. In the context of PG construction, automatic genome annotations usually comprise three procedures: (1) reference genes lift-over (projection), in which the annotation of the reference genome is mapped to other genomes from the same species (Shumate and Salzberg 2021); (2) ab initio gene prediction, in which gene models are detected based solely on their genomic sequences using pretrained models (Scalzitti et al. 2020); and (3) evidence-based predictions, in which homologies detected between input proteomic or transcriptomic sequences and the target genome sequence are used for the establishment of high-confidence gene models. Evidence-based predictions are highly dependent on the amount and quality of the provided evidence. We thus explored the effect of the quality of the provided evidence on the genome annotations of specific ecotypes and on the overall PG content. To this end, we used 3 sets of annotation evidence as inputs for PG construction with either the DN or the MTP approach: No evidence (i.e., based only on ab initio predictions and reference gene lift-over), General evidence (based on The Arabidopsis Information Resource [TAIR] reference proteome and a general collection of transcriptome data from 18 *A. thaliana* accessions), and HQ evidence (transcriptome and proteomes produced from all 8 ecotypes present in the PG). In all cases, the same genome assemblies (based on 50× sequencing data) and annotation procedures were used.

We examined the number of reference and nonreference genes detected in each PG. Although the number of reference genes was highly similar across data sets, the number of nonreference genes appeared to be strongly affected by the annotation evidence (supplementary table S9, Supplementary Material online). For both the MTP and DN approaches, the number of nonreference genes was considerably higher in the No evidence PGs compared with the General evidence PGs (MTP: 6,813 and 669 for No evidence and General evidence, respectively; DN: 11,457 and 4,565 for No evidence and General evidence, respectively). This indicates that although reference genes can be successfully detected using a lift-over procedure, the main benefit of using annotation evidence is the reduction in the number of nonreference genes that are predicted ab initio but lack protein/transcript support. A different trend was observed when comparing the PGs constructed using the General and HQ evidence PGs. Using the MTP approach, the number of nonreference genes was higher in the HQ evidence PG (1,777) than in the General evidence PG (669), but an opposite trend was observed when comparing the DN PGs (3,546 and 4,565 nonreference genes in HQ evidence and General evidence, respectively). We hypothesize that in both DN and MTP approaches, providing HQ evidence leads to an increase in the number of detected genes within novel (nonreference) genomic sequences. However, in the DN approach, this effect is masked by high rates of false gene detections in genomic regions with high homology to the reference when providing low-quality evidence. This results in an overall higher number of nonreference genes when using low-quality evidence. Because the MTP approach only annotates novel genomic regions, it is more robust to these false detections and hence the higher number of nonreference genes in the HQ evidence PG. This hypothesis is supported by the observation that the ratio of nonreference transcripts which could be reliably mapped to the reference genome (query coverage > 95%) was higher for the DN General evidence PG (39%) compared with the DN HQ evidence PG (27%), indicating that more of these genes reside in reference-like sequences and may result from false predictions.

### The Effect of Parameter Values and Choice of Software Tools

PG construction is a multistep procedure involving the application of various computational tools, each with its specific parameters and thresholds which need to be set by the user. However, it is often difficult to determine the optimal values for these parameters or even predict their effect on the constructed PG. We examined the effects of several such parameters which we expected to be particularly influential. First, we evaluated the effect of two parameters related to gene presence–absence detection in the MTP and IA approaches, termed the depth threshold (i.e., the minimal number of mapped reads required to label a given position as covered) and the coverage threshold (i.e., the percentage of the gene sequence labeled as covered required to assign the gene as present in a given accession). We found that within the commonly used range of values, gene presence–absence inference as well as the PG composition is not highly affected. Choosing parameter values outside of this range, however, could highly impact the inferences (supplementary note 5, Supplementary Material online and supplementary fig. S3, Supplementary Material online). Moreover, the exact sequencing depth and parameter values required for reliable gene presence–absence inference may depend on the quality of the sequencing data and the available assemblies and thus may differ between studies. It is therefore recommended that researchers first optimize their pipelines for the specific data sets at hand.

Another factor that may affect novel gene detection, as well as gene presence–absence calling in the IA and MTP pipelines, is the alignment software used for short-read mapping. Read mapping is applied at two different steps: (1) in the IA procedure, reads of each accession are iteratively mapped to the reference genome to detect nonreference (unmapped) reads, which are subsequently assembled into nonreference contigs; and (2) in both IA and MTP, once a nonreference sequence is detected and annotated, reads from each accession are mapped to the PG to determine the presence and absence of pan-genes in the input accessions, thus constructing the final gene PAV matrix.

We examined the effect of the read mapping algorithm at these two steps by constructing *A. thaliana* PGs from the same 50× sequencing data, using either of two commonly used tools: BWA (Li and Durbin 2009) and Bowtie2 (Langmead and Salzberg 2012). Using Bowtie2, 30% more reads were considered unmapped and thus detected as nonreference, leading to a 26% increase in the size of the nonreference PG and a 5-fold increase in the number of detected nonreference genes compared with BWA. These results are expected because Bowtie2 is known to map reads more restrictively compared with BWA. On the other hand, the choice of the read mapping algorithm was found to have a relatively modest effect on gene presence–absence detection, with only 0.3% of the genes affected by this factor. We therefore conclude that the choice of read mapping algorithm has a considerable effect on the results of the IA approach, mainly due to differences in detecting nonreference sequences. Gene presence–absence detection, performed in both the IA and MTP approaches, is affected to a much lesser degree (further details on these analyses are provided in supplementary note 7, Supplementary Material online and supplementary table S10, Supplementary Material online).

Finally, we examined the effect of the clustering “inflation” parameter used for detection of gene orthology within the DN approach. This parameter controls the level of cluster granularity in the underlying Markov clustering (MCL) algorithm (Li et al. 2003) and therefore may affect the size and composition of constructed PGs, as well as gene presence–absence inference for specific accessions. We repeated the orthology clustering and gene presence–absence inference steps using a range of values for this parameter and found that around the MCL default value, the effect of this parameter is modest (supplementary fig. S5, Supplementary Material online and supplementary note 8, Supplementary Material online). Here again, the use of extreme values resulted in a considerable effect on the number of nonreference pan-genes and PG composition.

### The Effect of Methodological Factors on PG Construction in Soybean

We tested the generality of the observations described above by comparing PGs containing eight cultivated soybean (*G. max*) accessions (supplementary table S2, Supplementary Material online), using different construction approaches and different genome assemblies (as detailed in supplementary table S1, Supplementary Material online). The soybean genome is approximately ten times larger and is considerably more repetitive and complex compared with the *A. thaliana* genome. Our results in soybean are in line with those observed in *A. thaliana*, but here, the differences between the various construction approaches were even more extreme (all results are detailed in table 1, supplementary tables S11–S14, Supplementary Material online, and supplementary fig. S2, Supplementary Material online).

First, we compared PGs constructed using the DN, MTP, and IA approaches from 50× short-read sequencing data and observed considerable differences in size, composition, and content (table 1 and supplementary fig. S2, Supplementary Material online). Specifically, the DN, MTP, and IA PGs contained a total of 70,898, 52,058, and 52,287 pan-genes, respectively. Of these, the number of nonreference pan-genes is 66 times and 37 times higher in the DN PG compared with the MTP and IA PGs, respectively (DN, 19,129; MTP, 289; and IA, 518). As observed in *A. thaliana*, the nonreference gene pools were highly dissimilar, with only 74 matched nonreference pan-genes, shared by the DN and MTP PGs. The PG compositions also differed substantially: although the MTP and IA PGs resulted in 98% core genes, only 54% core genes were obtained in the DN PG. We further compared the DN and MTP soybean PGs by considering the degree of disagreement in gene presence–absence calls. A total of 38,574 genes were found to be core genes by both the DN and the MTP procedures. However, when examining only the 13,269 pan-genes that are noncore in either of the PGs, we found that 49% of all presence–absence calls differed between the two PGs, with 98% of the genes displaying at least one discrepancy. Discrepancies were almost exclusively (>99%) of the DN−|MTP+ type.

We examined the effect of assembly quality on soybean PGs by constructing additional PGs from sequencing data subsampled to 20× depth as well as from previously published HQ genome assemblies. As expected, assemblies produced using 50× sequencing depth were superior to 20× assemblies in terms of contiguity (average N50 9,133 and 5,858, respectively) and completeness (average assembly size 811 Mb and 756 Mb, respectively). These assemblies are of lower quality compared with those obtained for *A. thaliana*, which can be explained by the higher complexity and repetitiveness of the soybean genome. Still, the high percentage of complete BUSCOs (>97% for all assemblies) suggests that gene prediction may be successfully performed on assemblies produced from as low as 20× sequencing data. For DN PGs, the total number of pan-genes increased as assembly quality improved, which can be explained by the large difference in assembly sizes between 20×, 50×, and HQ-assembly data sets. In addition, the percentage of core genes was affected by assembly quality (51%, 54%, and 40% for 20×, 50× and HQ assemblies, respectively). In contrast, and in line with the results observed for *A. thaliana*, the MTP PGs were highly similar across different assembly qualities (all with nearly the same number of pan-genes with 97–98% of core genes).

### Meta-analysis of Previously Published PGs

**Fig. 4.**
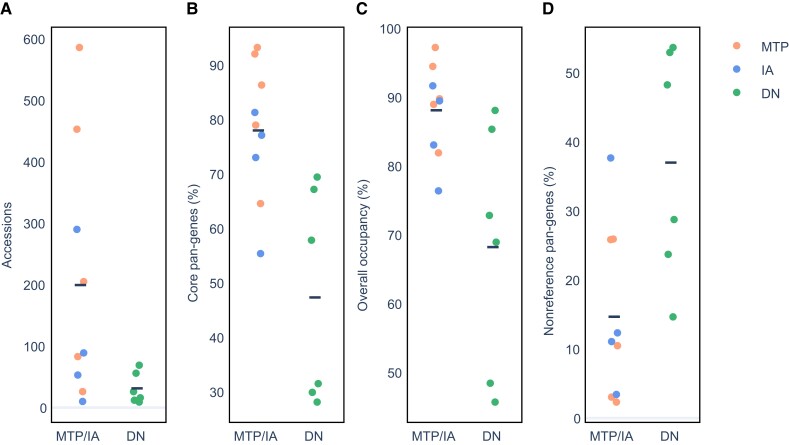
Meta-analysis of published plant PGs. Gene PAV matrices from 15 PG studies were curated, and four statistics were compared between MTP/IA and DN PGs. (*A*) Number of accessions. (*B*) Percentage of core pan-genes. (*C*) Overall percentage of gene occupancy. (*D*) Percentage of nonreference pan-genes. Horizontal lines represent the means of the compared groups.

To further assess the generality of our results across additional plant PGs, we conducted a meta-analysis of previously published data sets. To this end, we performed a literature search and obtained a total of 15 plant PG data sets for which a gene PAV matrix was available (table 3 and supplementary table S15, Supplementary Material online). Out of these, five studies applied variants of the MTP approach, four applied the IA approach, and six used the DN approach. For simplicity, in this section, we treat IA as a variant of the MTP approach and collectively refer to PGs constructed using either MTP or IA as MTP/IA. The number of accessions included in a PG varied considerably, ranging between 9 (*Brassica napus*) and 586 (tomato). Notably, the DN approach was never chosen for PGs including more than 70 accessions, whereas the MTP/IA approach was chosen for PGs of varying size (MTP/IA mean, 199 accessions; DN mean, 31 accessions; fig. 4*A*). This tendency of choosing different approaches for constructing PGs with respect to the number of sequenced accessions reflects the technical and computational challenges of assembling and annotating hundreds of plant genomes, as required by the DN approach.

**Table 3:** Statistics for Previously Published Plant PGs

Organism	Citation	Number of Accessions	Construction Approach	Total Pan-genes	Nonreference Pan-genes (%)	Overall Occupancy (%)	Core Pan-genes (%)
*B. napus*	Song et al. (2020)	9	DN	105,672	28.74	68.94	31.5
	Hurgobin et al. (2018)	53	IA	94,013	12.34	89.47	77.11
Rice	Zhao et al. (2018)	69	DN	42,580	23.71	72.82	57.84
	Wang et al. (2018)	453	MTP	48,099	25.92	81.92	64.56
*Brachypodium distachyon*	Gordon et al. (2017)	56	DN	61,158	52.93	45.7	28.12
Cucumber	Li et al. (2022)	12	DN	26,822	14.66	85.34	69.43
*Medicago truncatula*	Zhou et al. (2017)	16	DN	129,650	48.24	88.08	67.17
Maize	Hufford et al. (2021)	26	DN	103,032	53.65	48.44	29.9
Apple	Sun et al. (2020)	83	MTP	69,411	10.49	89.77	78.96
*Brassica oleracea*	Golicz et al. (2016a, 2016b)	10	IA	61,379	11.08	91.66	81.29
Eggplant	Barchi et al. (2021)	26	MTP	35,148	2.32	94.47	92.03
Pigeon pea	Zhao et al. (2020)	89	IA	55,512	3.42	76.39	55.38
Soybean	Torkamaneh et al. (2021)	205	MTP	54,533	3.04	97.23	93.22
Sunflower	Hübner et al. (2019)	287	IA	45,302	37.66	83.06	73.02
Tomato	Gao et al. (2019)	586	MTP	40,369	25.84	88.95	86.33

We compared the PG compositions across data sets by computing two metrics for each PAV matrix: (1) the percentage of core pan-genes, defined as those present in at least 95% of the accessions, and (2) overall occupancy, defined as the total percentage of gene presence across all pan-genes and accessions. Although the percentage of core pan-genes is a common and biologically meaningful metric for assessing PG composition, it requires the arbitrary choice of a presence cutoff (in this case 95%), whereas the occupancy measure is a more global metric. Nevertheless, the two metrics show high linear correlation (*R*^2^ = 0.88, supplementary fig. S4*A*, Supplementary Material online). We observed a substantial difference in PG composition between PGs constructed using the MTP/IA approach compared with those constructed using the DN approach, with MTP/IA PGs showing significantly higher percentages of core genes (MTP/IA mean, 77.98%; DN mean, 47.33%; two-sided *t P* = 0.002; fig. 4*B*), as well as higher overall occupancy (MTP/IA mean, 88.1%; DN mean, 68.22%; two-sided *t P* = 0.009; fig. 4*C*). We note that the association between the number of accessions and the two PG composition metrics was very low (*R*^2^ = 0.1 and *R*^2^ = 0.05 for the percentage of core genes and overall occupancy, respectively; supplementary fig. S4*B*–*C*, Supplementary Material online). This indicates that the observed differences are likely the result of the construction approach rather than the number of accessions and is consistent with the observed differences in *A. thaliana* and soybean PG compositions reported above. We further found that MTP/IA PGs have significantly lower percentage of nonreference pan-genes (MTP/IA mean, 14.68%; DN mean, 36.99%; two-sided *t P* = 0.01; fig. 4*D*). Together, the findings of the meta-analysis suggest that the higher numbers of nonreference genes detected by the DN approach is a general phenomenon, not restricted to the PGs of *A. thaliana* and soybean examined above. It should be noted, however, that some of the observed differences may derive from biological phenomena related to the choice of the accessions, life history traits, and genomic features of the various species such as recent polyploidy events.

## Discussion

A central aim for constructing plant PGs is to explore the genomic diversity underlying phenotypic variation existing within a plant lineage. Establishing extensive PG data sets is expected to enable applications such as the detection of novel resistance genes and association of gene presence–absence distribution with desired traits. As the usage of pan-genomics for the study of plant diversity becomes more feasible and common, better understanding of the involved procedures is crucial. The results of our study suggest that a plethora of methodological and technical factors affect the construction of a gene-based PG and these should be determined and accounted for throughout the analysis. Despite the increasing number of publications focused on the construction and exploration of plant PGs, different PG projects have applied considerably different methods and computational pipelines, with no well-established standards for the construction procedure. This emphasizes the importance of better understanding the consequences of the applied methodological choices that need to be made throughout the construction process.

The direct comparison between different PG construction procedures required the development of novel methods allowing for pairwise comparisons of PGs. We have suggested several global measures for summarizing PG compositions and structure (e.g., the total number of pan-genes, the ratio of nonreference pan-genes, the proportions of core pan-genes, and the overall gene occupancy), as well as methods for direct comparison of the gene content of two PGs. Such measures can be applied in future studies for the evaluation of PG quality, as they provide means for examining the two main benefits of a PG analysis: detection of nonreference genes and insight on intraspecific gene content diversity.

Several past publications predicted that various methodological factors may affect the results of PG construction procedures (Golicz et al. 2020; Hu et al. 2020; Lei et al. 2021). Specifically, it is probably not surprising that different construction approaches lead to considerably different PGs. It was previously suggested that the DN and MTP (or IA) approaches are complementary (Golicz et al. 2020) and that a hybrid approach may be applied. In practice, however, researchers often choose one approach over the others, taking into consideration features such as the available resources and the size of the diversity panel. Notably, all gene-based PG construction approaches result in a set of pan-genes and their inferred presence–absence matrix. As demonstrated here, the choice of the construction approach affects both these central outputs and should thus be chosen in an informed manner.

Our comparisons of the most common PG construction approaches revealed considerable differences in the resulting PGs. We found that the sets of nonreference genes show very partial overlaps between PGs constructed from the same data but using different procedures, with the great majority of genes detected by one of the approaches only. Moreover, even for pan-genes included in both PGs, numerous discrepancies exist regarding presence–absence inferences in specific accessions. We generally observed that the DN approach leads to larger pools of nonreference genes, whereas the MTP and IA approaches more readily determine genes to be present in specific accessions. This leads to marked differences in the reported PG composition. For example, PGs constructed using the DN approach typically display lower ratios of core genes compared with MTP PGs.

We also assessed the effect of sequencing depth and the quality of the genome assembly on the constructed PG. As expected, lower sequencing depth resulted in less complete and contiguous assemblies, whereas the availability of long reads improved assembly quality. However, in the case of gene-based PGs, the effect on the resulting PAV matrix and set of pan-genes is rather modest (e.g., fig. 3*B*). This could be explained by the relative ease by which coding regions can be assembled compared with repetitive and otherwise low-complexity intergenic regions. Moreover, in case the MTP construction approach is used, it is sufficient that a genomic region containing a gene is adequately covered to call that gene present, without the need that the relevant genomic region is assembled into contigs. Thus, although the exact sequencing depth required for PG construction may be species specific and depend on multiple genomic characteristics, our observations suggest that data sets with low sequencing depth produce gene-based PGs with very similar compositions compared with those with higher depths, at least if only short-read data are used. This observation is especially important due to the abundance of sequencing data from past genome resequencing projects which might be “recycled” for the purpose of PG construction. Another methodological factor that was shown to impact PG construction is the quality of sequences used as annotation evidence. Our results indicate that obtaining a HQ set of transcript and protein evidence is highly beneficial, especially for reducing the amount of false gene detection. More generally, we find that the annotation step presents one of the main challenges in PG construction procedures, regardless of the applied approach. One possible way to overcome this challenge is to obtain several HQ annotations based on chromosome-level genome assemblies and then project and consolidate gene models onto the PG assemblies (Walkowiak et al. 2020). Another useful practice is to reannotate the reference sequence using the same annotation procedure applied when constructing the PG. Comparing the annotation result with the published reference annotations can aid in the evaluation of the accuracy and completeness of the PG annotation.

Recent improvements in third-generation sequencing technologies are revolutionizing the field of genomics by providing long-range information. This, together with advancements in assembly algorithms, rapidly increases the availability of reference-quality genome assemblies for many plant species. Moreover, long-read RNA-seq allows for full transcript sequencing, which can be used as valuable annotation evidence. However, our results indicate that even if such HQ genome assemblies and transcriptomic data are available, other methodological factors such as the construction approach are expected to strongly affect the resulting PG composition.

It is very likely that many other methodological factors, not explored in this study, affect PG construction to some extent. Furthermore, interactions between methodological factors may be hard to predict, adding another layer of complexity. Implementations of any construction approach usually consist of multiple decisions related to the choice of software tools (e.g., for genome assembly and scaffolding, gene prediction, and read mapping) and specific parameter values, which may introduce discrepancies. Rigorous research of these factors may not always be feasible. One example is the choice of the genome assembler, which is known to have a major effect on the completeness, contiguity, and accuracy of assemblies (Dmitriev et al. 2022). Because the differences between genome assemblers have been extensively studied before, and due to the large parameter-space specific to each assembler, we did not analyze this factor here and refer the readers to previous studies (Cherukuri and Janga 2016; Jayakumar and Sakakibara 2019). Nevertheless, we observe some general trends when comparing DN with MTP and IA PGs produced using different implementations of the pipelines, which suggests that implementation details may have a subtler effect than the construction approach itself.

A major challenge encountered when evaluating the performance of different PG construction procedures is the lack of “ground-truth” data to which results can be compared. This limits our ability to assess performance using measures such as specificity, sensitivity, and accuracy. Various indirect measures based on comparisons to known sequences in public databases could provide general performance estimates. However, a more meticulous analysis will require the development of benchmarking data sets of carefully curated PGs or devising dedicated PG simulation software tools, which could reliably emulate the underlying processes that generate intraspecific diversity (Stephens et al. 2016; Ferrés et al. 2020). Likewise, high throughput methods for biological validation of sequence presence–absence calls in multiple genomes can significantly increase our ability to assess the reliability of PG construction procedures.

Despite being unable to directly assess the quality of the results obtained using different PG construction procedures, the observations of this study allowed us to formulate several general guidelines that could aid future researchers in making methodological decisions. First, with regard to the choice of the construction approach, the DN approach should be favored in case the main goal of the study is to obtain a large pool of candidate nonreference genes. This could be useful when phenotypes of interest are known to occur in the population of a species but not in the reference accession. The DN approach is also recommended when several HQ genome assemblies and annotations are available or when the study also includes analyses based on a PG graph. Unfortunately, due to the high sequencing and computational demands imposed by the DN approach, it might not always be feasible. In such cases, or in studies where the main focus is on the variation of gene presence–absence within large populations (e.g., in the context of association studies), the MTP and IA approaches may be advised. We suggest that the MTP approach should be favored over the IA approach because it allows the analysis of nonreference sequences in their genomic context. However, this choice would depend on the genome size of the studied organism and the number of sequenced accessions. When the computational resources available for the study are limited, the IA approach could prove to be a valuable alternative.

A common decision that researchers have to make when planning a PG project is whether to generate deep sequencing data for a small number of accessions or to sequence many accessions to lower depths, thereby balancing between the quality of specific genomes and the degree of genetic diversity covered in the data set. Based on our results, it appears that when constructing a gene-based PG, especially with the MTP and IA approaches, sequencing depth may be kept to the minimum required for successfully assembling the gene space. A simple rule of thumb may be that one should use the sequencing depth required to achieve an assembly with at least 95% complete BUSCOs. Even when genome assemblies of satisfying quality are available, the process of gene annotation still poses a considerable challenge. Our results emphasize the need for obtaining HQ annotation evidence, and thus, we recommend that substantial resources are allocated to producing relevant data sets, preferably based on transcriptomic sequences obtained from the same accessions included in the PG.

Finally, a general recommendation for researchers constructing gene-based PG is to test the robustness of their results to methodological factors, for example, by applying multiple construction approaches. In addition, the development of hybrid approaches which could incorporate advantages from the DN. MTP, and IA procedures may be highly beneficial, although it requires further research.

Pan-genomics is gradually becoming a standard approach for studying genomic diversity within species. For plant genomes, such analyses are still in their infancy and new methods are actively being developed and applied. In general, we expect that the increasing availability of sequencing data, especially long reads, will allow researchers to abandon older techniques in favor of those that depend on multiple HQ genome assemblies. One such approach is based on the concept of PG graphs (Marschall et al. 2018; Rakocevic et al. 2019; Tao et al. 2020). To date, however, gene-based and graph-based PGs are constructed separately, using completely different procedures and are aimed for different analyses (Liu et al. 2020; Qin et al. 2021; Li et al. 2022): PG graphs are mainly used to identify and genotype structural sequence variation, whereas gene-based procedures such as the DN, MTP, and IA approaches studied here are used to identify the set of genes found within a species and their variation among accessions. A recent study reports the construction of a graph-based human PG from 47 diverse assemblies (Liao et al. 2023). The constructed graph was utilized to aid in gene annotation but was not directly used for gene-related analyses. Although this is an important step toward the use of graph-based techniques for gene presence–absence detection, considerable research is still required to overcome some challenges and limitations related to variation graph algorithms (Sherman and Salzberg 2020). Therefore, we expect that better understanding of gene-focused methods will be of great importance when more sophisticated techniques combining them with graph-based algorithms are developed.

Regardless of the specific methodology, obtaining solid understanding of the various factors affecting the constructed PGs is of high importance. The consistency of our observations across data sets, species, and studies indicates that methodological effects are not limited to specific input data or implementations of the construction procedures. Rather, they represent a general phenomenon that needs to be addressed, and researchers drawing biological conclusions from PG studies should be made aware of these effects.

## Materials and Methods

### Data for PG Construction

Short Illumina and long PacBio reads in FastQ format were downloaded from the European Nucleotide Archive (ENA) for *A. thaliana* and from the Genome Sequence Archive (GSA) for soybean, based on accession numbers as detailed in supplementary table S2, Supplementary Material online. We then randomly subsampled the FastQ files to match the required sequencing depth by keeping only the desired number of reads from the original files.

The reference genome, gene annotation, transcript, coding sequence, and protein sequences for *A. thaliana* Col-0 ecotype version TAIR10.45 were downloaded from Ensembl Plants. Additional HQ genome assemblies and transcriptomes for seven *A. thaliana* ecotypes were obtained from the “1001 genomes” website, from the MPIPZ project (Jiao and Schneeberger 2020). Additional transcript sequences were obtained from the WTCHG project (Gan et al. 2011).

The soybean Williams82 reference sequences and annotation (assembly version 4, annotation version 1), as well as transcriptomes of two *G. max* accessions (Lee and ZH13) and two *Glycine soja* accessions (W05 and PI483463), were downloaded from SoyBase. Genome assemblies for an additional seven *G. max* accessions were downloaded from GSA (see supplementary table S2, Supplementary Material online).

### PG Construction

PGs were constructed using three computational pipelines (DN, MTP, and IA) implemented in the software package Panoramic v1.2.1 (Glick and Mayrose 2021). These pipelines contain the following steps:

**Reads preprocessing (DN, MTP, and IA):** short sequencing reads were filtered and trimmed using Trimmomatic v0.39 (Bolger et al. 2014) with parameters “SLIDINGWINDOW:5:15 MINLEN:40.” Paired-end reads were merged using Flash v1.3.0 (Magoč and Salzberg 2011) with parameters -m 10 -x 0.2.

**Read mapping (MTP and IA):** in the IA approach, short reads were mapped to the reference genome and to the PG using Bowtie2 v2.5.1 (Langmead and Salzberg 2012) . In the MTP approach, short reads were mapped to the PG for gene presence–absence detection using BWA v0.7.17 (Li and Durbin 2009) with the *mem* algorithm and default parameters. Long reads were mapped using Minimap2 v2.17 (Li 2018) with parameters -ax asm20.

**Extraction of unmapped reads (IA):** using samtools v1.15.1 (Li et al. 2009), unmapped reads were extracted using the command “samtools view -h -e ‘flag.unmap || mapq <= 10 || [NM] >= 8 || qlen <= 80'” and converted to fastq format using the command “samtools fastq”, When both reads of a pair were determined as unmapped, pairing was retained.

**Genome assembly and quality assurance (DN, MTP, and IA):** preprocessed whole genome data (DN and MTP) or unmapped reads (IA) were assembled into contigs using SPAdes v3.13.2 (Bankevich et al. 2012) (*A. thaliana*) or Minia (Chikhi and Rizk 2013) (soybean) with default parameters. Contigs shorter than 300 bp were discarded, and the remaining contigs were scaffolded into pseudomolecules (DN and MTP only) based on the reference sequence, using RagTag v1.0.1 (Alonge et al. 2019). Assembly quality was assessed using QUAST v5.0.2 (Gurevich et al. 2013) and BUSCO v5.0.0 (Waterhouse et al. 2018) with lineages brassicales_odb10 for *A. thaliana* and fabales_odb10 for soybean.

**Detection of nonreference genomic sequences (MTP and IA):** The procedure begins with the PG equivalent to the reference sequence. At each step, the genome assembly from a single accession is mapped to the PG, using Minimap2 v2.17 (Li 2018) with parameter -ax asm5, and unmapped sequences (contigs or parts of contigs) are extracted and added to the PG using a dedicated python script. This step is repeated until all genomes have been mapped. Unmapped sequences shorter than 300 bp for *A. thaliana* and 500 bp for soybean were discarded.

**Gene annotation (DN, MTP, and IA):** whole genome assemblies (DN) or the nonreference section of the PG (MTP and IA) were annotated by integrating multiple steps to produce candidate gene models. First, repetitive sequences were masked using EDTA (Ou et al. 2019). Reference genes were lifted-over masked genomes using Liftoff v1.6.1 (Shumate and Salzberg 2021) (DN only). For ab initio predictions, we used Augustus v3.4.0 (Stanke and Waack 2003), SNAP v2013_11_29 (Korf 2004), and GlimmerHMM v3.0.4 (Majoros et al. 2004) (*A. thaliana* only). For *A. thaliana*, the relevant pretrained models provided with the software tools were used, whereas for soybean, Augustus and SNAP were first trained based on single-copy BUSCOs detected in the reference assembly. We used PASA v2.4.1 (Haas et al. 2008) to create gene predictions based on transcript sequence homology. For “standard evidence” PGs, the input transcripts were those from the WTCHG set (*A. thaliana*) or the GSA set (soybean), whereas for “high-quality evidence” PGs, we used transcripts from the MPIPZ set (*A. thaliana*). To reduce evidence redundancy and run times, transcripts were first clustered using MMseq2 v13.45111 (Steinegger and Söding 2017) with the command “mmseqs easy-linclust”, and a single representative transcript was taken from each cluster. Gene models were generated from reference protein sequences using GenomeThreader v1.7.1 (Gremme et al. 2005). Gene predictions derived from lift-over, ab initio, and evidence-based analyses were combined into gene models using EvidenceModeler v1.1.1 (Haas et al. 2008), with weights set to 20 for lift-over, 10 for transcript alignments, 5 for protein alignments, and 1 for all ab initio outputs. Finally, low-confidence gene models were discarded. To this end, we computed the annotation edit distance (AED; Eilbeck et al. 2009) as a confidence score indicating the support of gene models by transcript and protein evidence. Gene models derived solely from ab initio predictions (AED = 1) and those with protein products shorter than 50 amino acids were then discarded.

**Orthology clustering (DN):** protein products derived from candidate gene models were clustered into orthology groups using OrthoFinder2 v2.5.1 (Emms and Kelly 2019). The resulting orthogroups frequently contain multiple genes from the same accession, which are possibly paralogous. Thus, orthology groups were further split into subclusters using the Maximum Orthogonal Weight Partitioning algorithm (Zheng et al. 2011) to ensure they represent pan-genes rather than gene families. Further details are provided in Glick and Mayrose (2021) (see supplementary note 1, Supplementary Material online and supplementary fig. S2, Supplementary Material online therein).

**Gene presence–absence detection based on reads coverage (MTP and IA)**: the coverage of exons for each gene model was computed based on read mapping, using the command “bedtools coverage -hist -sorted” from bedtools v2.30.0 (Quinlan and Hall 2010). A gene was considered present in an accession if at least 40% of its exonic sequence were covered with a depth of three or higher.

### Pairwise Matching of Nonreference Protein Sets

Sets of nonreference protein sequences were compared by performing reciprocal protein Blast searches (Camacho et al. 2009), with each protein set used both as the query and the database. Bitscores were normalized to account for sequence length bias by applying the procedure described by Emms and Kelly (2015), after which the bidirectional bitscore for each protein pair was calculated as the average normalized bitscore from the two reciprocal Blast runs. In cases where no hits between proteins were found, a bitscore of zero was assumed. Next, a full bipartite graph of all Blast hits was generated and the maximum weight matching was detected using the Python package Networkx (Hagberg et al. 2008). Matches with a reciprocal bitscore lower than 100 were discarded, and only the remaining protein pairs were considered matched.

### Analysis of Nonreference Gene Sequences

Transcript sequences were mapped to genomic sequences using Minimap2 (Li 2018) with parameters -x splice:hq -uf. The query coverage of matches was calculated based on the output PAF format file as number of residue matches/query sequence length. Protein sequence mapping was performed using BlastP (Camacho et al. 2009) with *E*-value threshold set to 10^−5^ and otherwise default parameters. The query coverage was calculated as alignment length (excluding gaps) divided by the query length.

To profile the expression of nonreference genes, RNA-seq reads derived from multiple studies (supplementary table S15, Supplementary Material online) were downloaded from ENA. All paired-end files were concatenated, and then 10% of read pairs were randomly selected. RNA-seq reads were mapped to transcript sequences of nonreference genes using BWA v0.7.17 (Li and Durbin 2009) using default parameters. Unmapped reads (SAM flag 4) and those with mapping quality lower than 20 were discarded using the command “samtools view -F 4 -q 20” (Li et al. 2009). The number of reads mapped to each transcript was counted and divided by the transcript length to compute RPKM.

### Meta-Analysis of Previously Published PGs

Gene PAV data for each study were downloaded or obtained through personal communication, as detailed in supplementary table S16, Supplementary Material online. All files were processed using data set–specific functions and simplified into a uniform format where gene presence and absence are coded as 1 and 0, respectively.

## Supplementary Material

evad121_Supplementary_Data

## Data Availability

The Panoramic code used for PG construction can be found at https://github.com/MayroseLab/Panoramic. All code used for comparison and analysis of PGs can be found at https://github.com/MayroseLab/PGCM, as Python scripts and Jupyter Notebooks. Raw sequencing data are available from ENA under accession numbers as detailed in the relevant supplementary tables, Supplementary Material online. PGs, genome assemblies, and annotations were deposited at DRYAD under DOI doi:10.5061/dryad.69p8cz93w (https://datadryad.org/stash/dataset/doi:10.5061/dryad.69p8cz93w).
