# Supplementary material for: The Effect of Methodological Considerations on the Construction of Gene-Based Plant Pan-genomes
Source: Genome Biol Evol. 2023 Jul 4;15(7):evad121. doi: 10.1093/gbe/evad121 (PMC10340445; doi:10.1093/gbe/evad121)
Supplement: evad121_Supplementary_Data [file evad121_supplementary_data.zip › Supplementary figures.docx]

# Supplementary figures for: On the vulnerability of plant pan-genomes to methodological considerations

### Figure S1


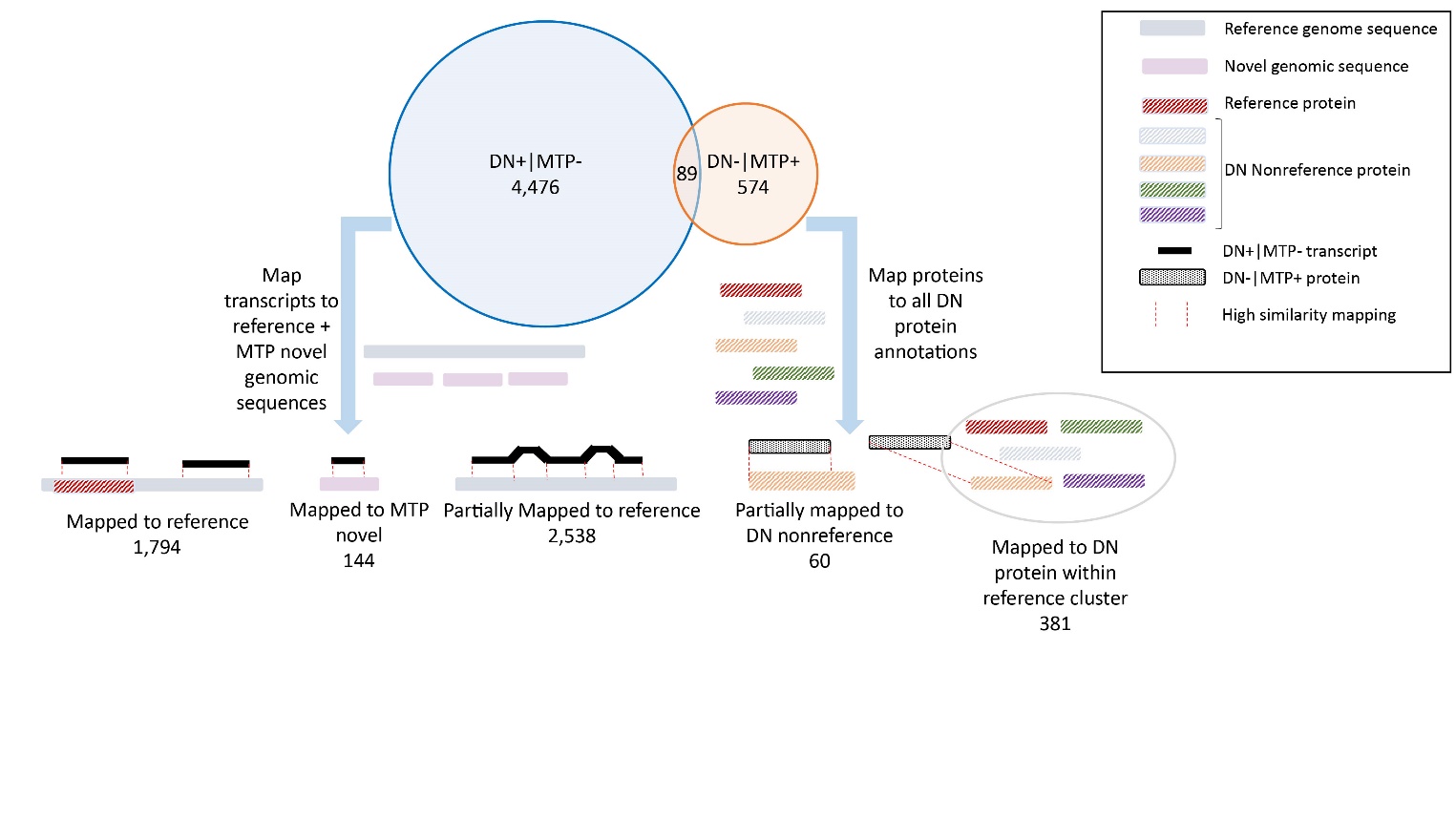


### Figure S2


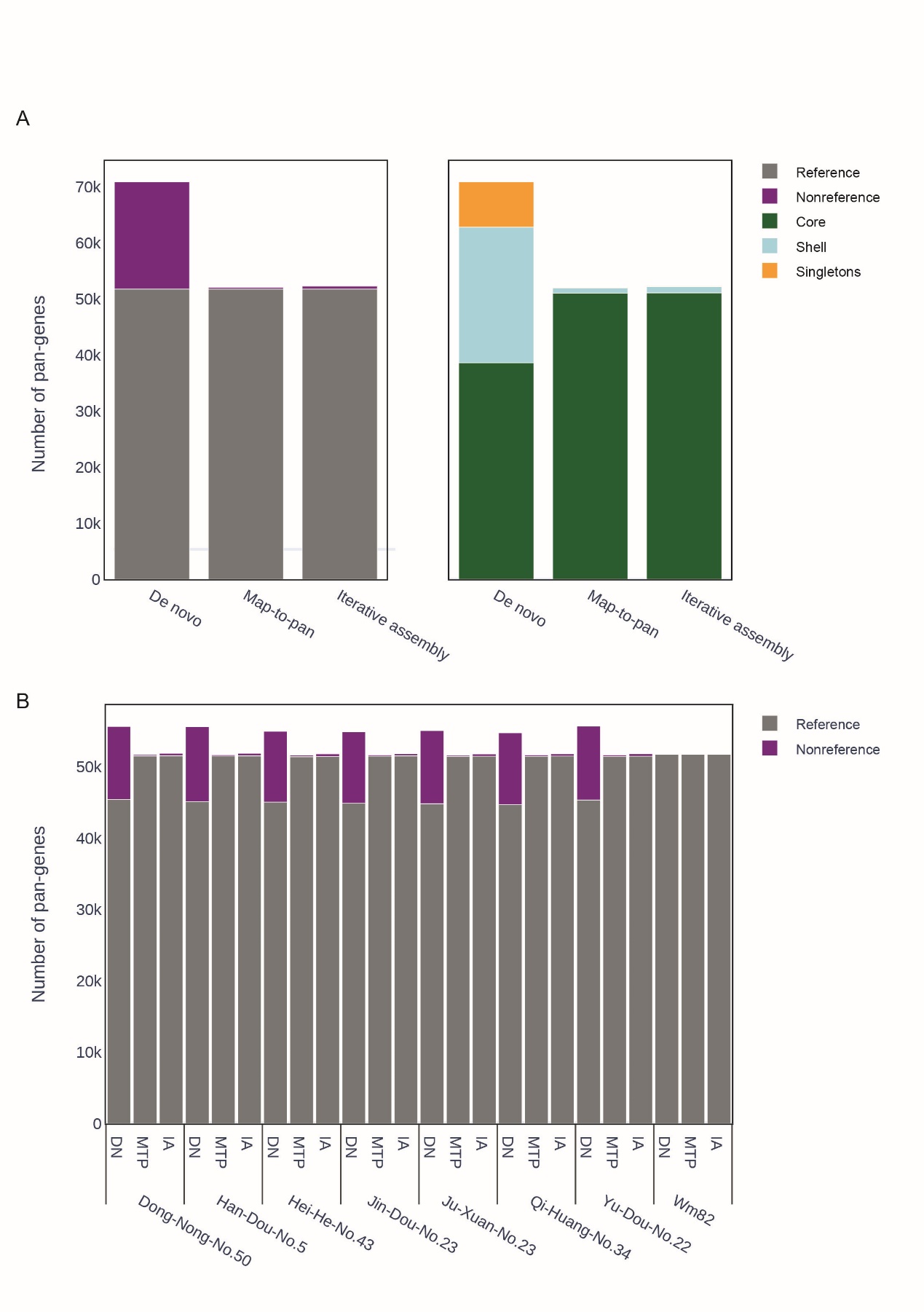


### Figure S3


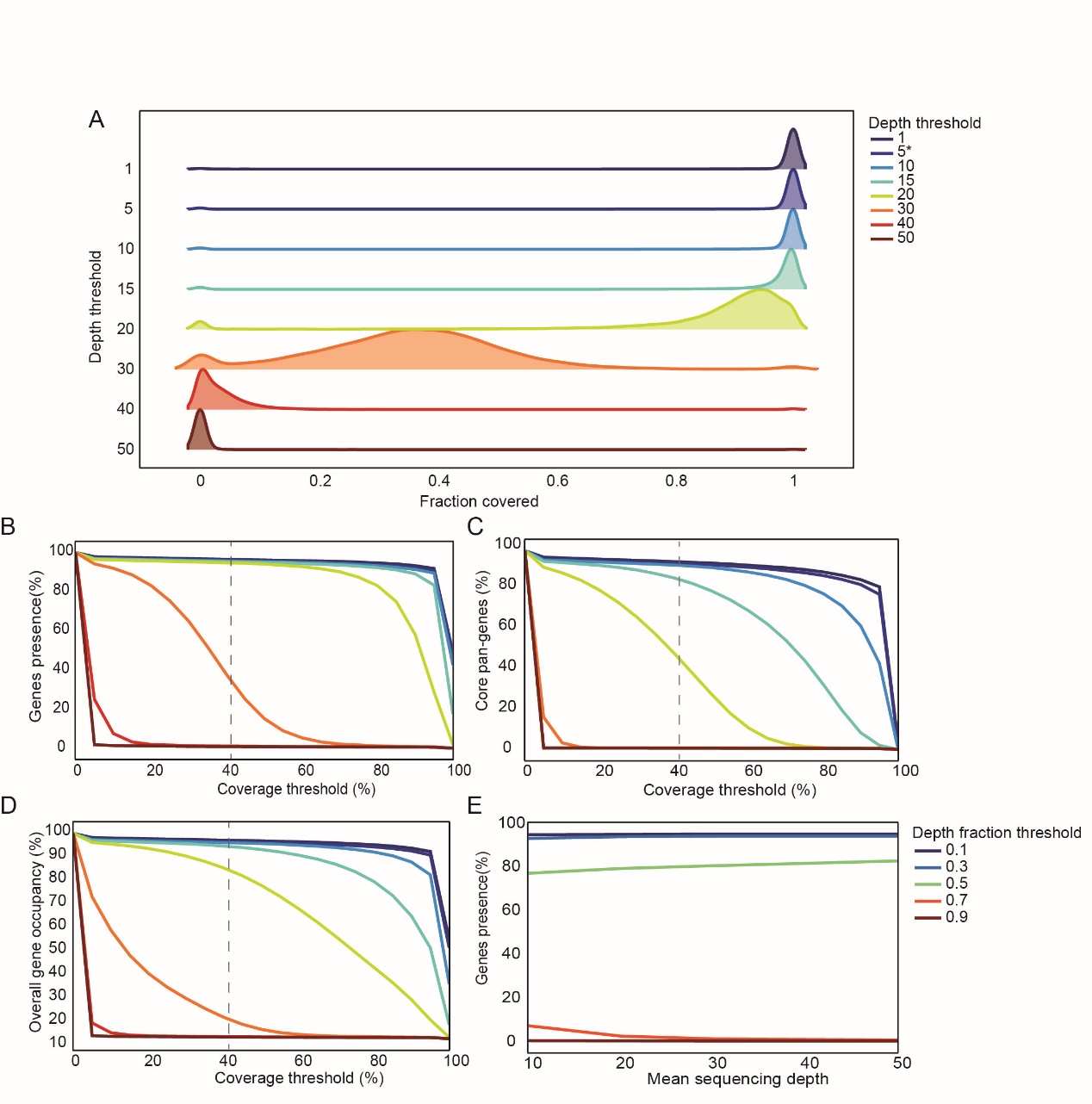


### Figure S4


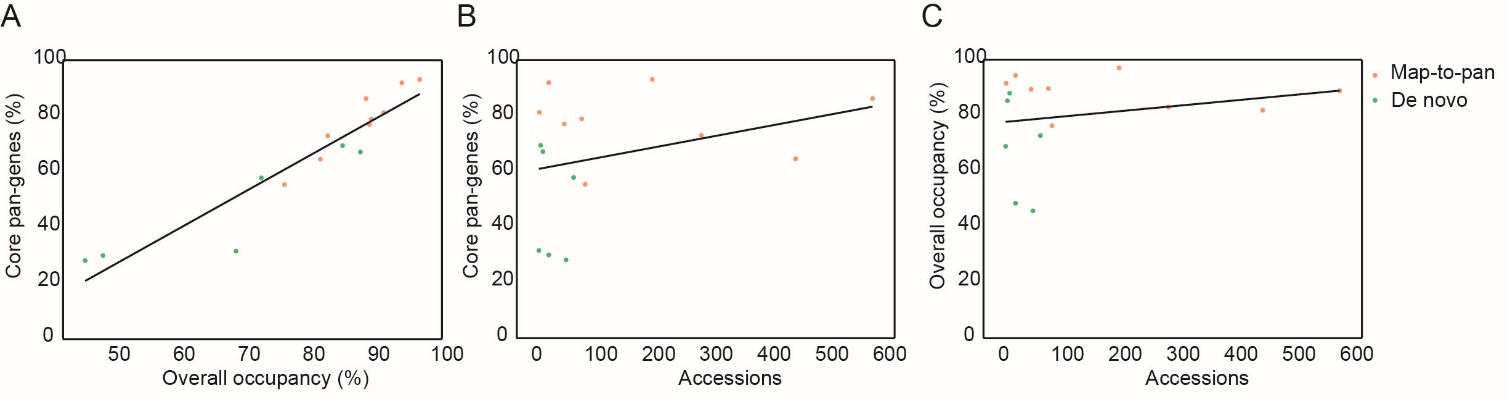


### Figure S5


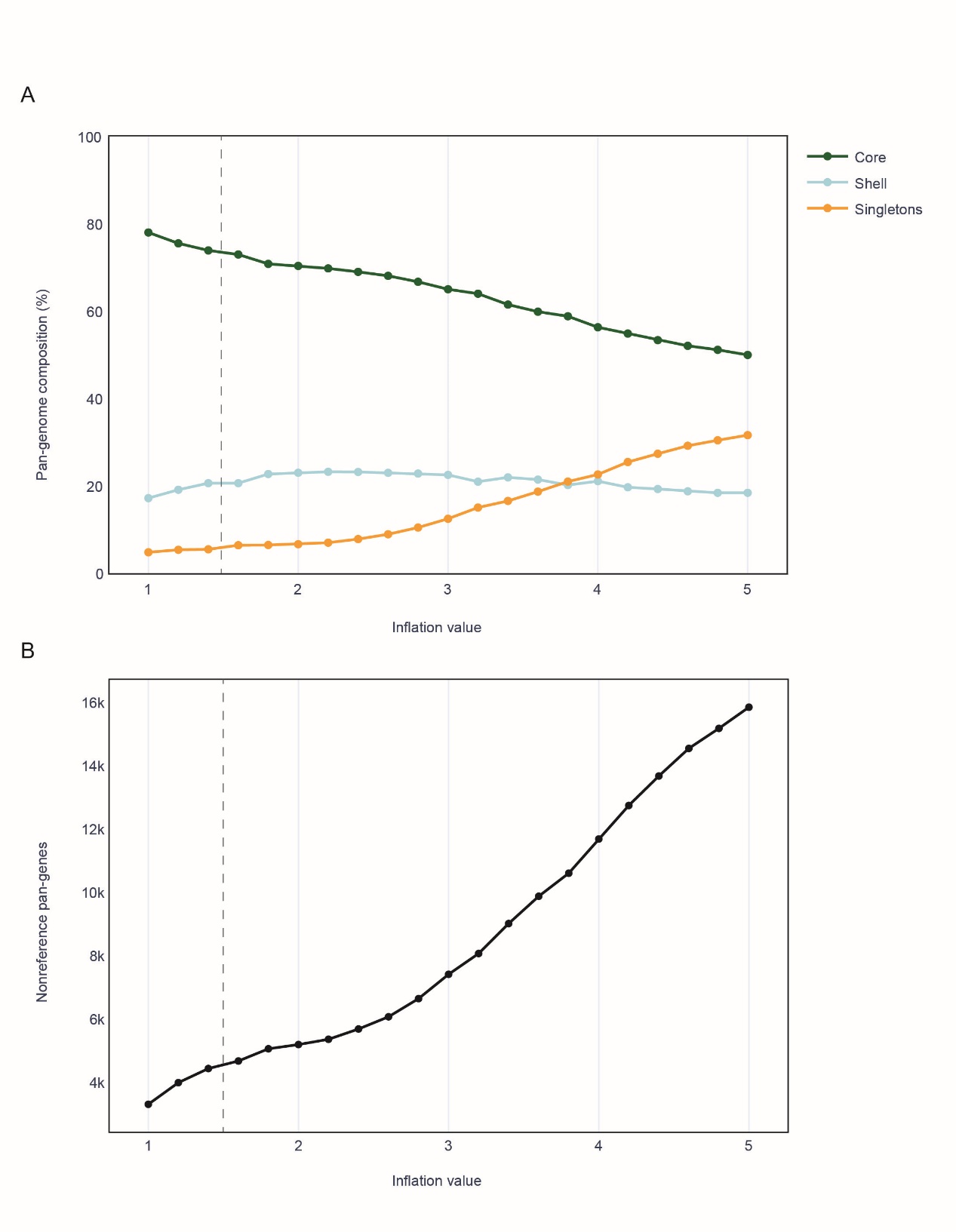


## Supplementary figure legends

**Figure S1. Analysis and classification of DN+|MTP- and DN-|MTP+ nonreference pan-genes in *A. thaliana*.** The Venn diagram shows the overlap between the sets of nonreference pan-genes detected by the DN and MTP pan-genome construction approaches. Transcript sequences of DN+|MTP- pan-genes (black bars) were mapped to the reference genome sequence (grey bars) and the MTP nonreference sequences (purple bars). DN-|MTP+ genes were analyzed by mapping their protein sequences (dotted bars) to reference proteins (red striped bars) and to DN nonreference proteins (striped bars).

**Figure S2. Comparison of soybean pan-genomes constructed using the DN, MTP, and IA approaches.** The same input data were used for constructing three pan-genomes containing eight cultivated soybean accessions, using the DN, MTP, and IA approach. **(A)** The overall pan-genome sizes and compositions – number of reference (grey) and nonreference (purple) pan-genes, as well as core (green), shell (light blue), and singletons (orange). **(B)** Number of reference and nonreference pan-genes detected by the three approaches in each ecotype.

**Figure S3. Analysis of *A. thaliana* gene presence-absence detection thresholds in the Map-to-pan approach. (A)** Distributions of the fractions of genes covered by mapped reads in a single *A. thaliana* ecotype (An-1) using different depth thresholds on 50× sequencing data. **(B)** The percentage of genes determined as present as a function of the coverage threshold, using different depth thresholds on 50× sequencing data of a single *A. thaliana* ecotype (An-1). **(C)** The percentage of core pan-genes as a function of the coverage threshold in an *A. thaliana* pan-genome containing eight accession, using different depth thresholds on 50× sequencing data. **(D)** The overall gene occupancy as a function of the coverage threshold in an *A. thaliana* pan-genome containing eight accession, using different depth thresholds on 50× sequencing data. **(E)** The percentage of genes determined as present as a function of the sequencing depth using different depth fraction thresholds on 50× sequencing data of a single *A. thaliana* ecotype (An-1). The vertical dashed grey lines in B-D and the star (*) in the depth threshold legend indicate the default values used in Panoramic.

**Figure S4. Meta-analysis of published plant pan-genomes**. Fifteen published plant pan-genomes constructed using the Map-to-pan (orange) and De novo (green) approaches were analyzed. **(A)** Correlation between two pan-genome composition measures: the percentage of core pan-genes (present in 95% of pan-genome accessions), and the overall gene occupancy. **(B)** Correlation between the number of accessions included in a pan-genome and the percentage of core pan-genes. **(C)** Correlation between the number of accessions included in a pan-genome and the overall gene occupancy.

**Figure S5. Analysis of the effect of the MCL inflation parameter in the *A. thaliana* De novo approach. (A)** The proportions of core, shell, and singleton pan-genes using different inflation values on the same sets of protein sequences. **(B)** The number of nonreference pan-genes detected using different inflation values on the same sets of protein sequences. The vertical dashed grey lines indicate the default inflation value used in Panoramic.
