# Supplementary material for: The Effect of Methodological Considerations on the Construction of Gene-Based Plant Pan-genomes
Source: Genome Biol Evol. 2023 Jul 4;15(7):evad121. doi: 10.1093/gbe/evad121 (PMC10340445; doi:10.1093/gbe/evad121)
Supplement: evad121_Supplementary_Data [file evad121_supplementary_data.zip › Supplementary notes.docx]

# Supplementary information for: The effect of methodological considerations on the construction of gene-based plant pan-genomes

## Supplementary note 1 – comparison between the MTP and IA pan-genomes in *A. thaliana*

We constructed pan-genomes from the same 50x sequencing data of eight A. thaliana accessions, using either the MTP or the IA approaches. This resulted in different pan-genomes (see Table 1 in the main text), which were further compared and investigated.

Both construction approaches involve the detection of nonreference genomic sequences, which are subsequently annotated. The amount nonreference genomic sequences detected by the MTP approach (111 Mb) was 11% higher than that detected by the IA approach (100 Mb). Furthermore, the IA nonreference sequences were, on average, shorter compared to those obtained using the MTP approach (IA: 659 bp; MTP: 4,604 bp). 72% of the IA nonreference sequences could be mapped with high confidence to MTP nonreference sequences.

Despite the larger pool of nonreference sequences detected by the MTP approach, the number of nonreference pan-genes detected by the IA approach (959) was 45% larger than that detected by the MTP approach (663). Out of these, 258 pan-genes were detected by both approaches, and therefore the majority of pan-genes was only detected by either approach. We further found that the protein products of IA nonreference genes were on average 19% shorter than those of the MTP approach (IA: 217 aa; MTP: 258 aa). This may be explained by the previous observation that nonreference genomic sequences in the IA approach are generally shorter, which may lead to truncated gene annotations.

Next, we compared the gene PAV matrices derived from each of the pan-genomes. Pan-genes present in only one of the pan-genomes were ignored, leaving 27,295 reference and 258 nonreference pan-genes. Out of these 27.553 pan-genes, 25,505 were core genes (i.e. were inferred to be present in all eight accessions) in both the MTP and the IA pan-genomes, and were thus ignored in subsequent steps. Within the remaining 2,048 pan-genes, 502 (24%) contain at least one PAV discrepancy between the two pan-genomes. Out of the total 14,336 presence-absence calls across these pan-genes, 871 (6%) were in disagreement between the IA and MTP PAV matrices, with 465 being of the IA+|MTP- type and 406 being of the IA-|MTP+ type.

## Supplementary note 2 – analysis of DN+|MTP- nonreference pan-genes in *A. thaliana*

We investigated the methodological causes for the occurrence of DN+|MTP- pan-genes by mapping their transcript sequences to the reference genome and to the novel genomic sequences detected by the MTP approach. Based on the mapping results, DN+|MTP- pan-genes were classifying as:

1. Completely mapped to the reference genome
2. Partially mapped to the reference genome
3. Mapped to the MTP novel sequences

(also see Supplementary Figure S1).

If a DN+|MTP- transcript can be completely mapped to the reference genome it means that it is coded in a genomic region that is highly homologous to the reference and therefore not detected as novel by the MTP approach and not processed in the MTP annotation step. The transcript may map to a region marked as intergenic by the reference annotation, which can result from either gene emergence from previously noncoding regions in one of the annotated ecotypes, over-annotation by the DN pipeline, or under-annotation of the reference. Owing to the high quality of the reference annotation, the first two options appear more plausible. Alternatively, a DN+|MTP- transcript may successfully map to a reference genomic region coding for a reference gene, but translate into a highly different protein sequence. This can occur when the genomic sequence in one of the ecotypes contains a mutation that substantially modifies the protein sequence (e.g. frame-shift or stop codon loss/gain). We found that 1,794 DN+|MTP- (40% of the total DN+|MTP- pool) transcripts could be completely mapped to the reference genome (query coverage > 95%). Out of these, 38% overlap with reference genes (coordinate overlap of gene features > 50%), whereas 62% map to reference intergenic regions.

In other cases, a DN+|MTP- transcript only partially maps to the reference sequence. Such cases indicate that the gene is annotated in a region containing a combination of reference-homologous and non-reference-homologous sequences. If the stretches of nonhomologous sequences shorter than a certain cutoff (300 bp in this case), then they will be ignored in the MTP annotation process. We found that 2,476 (55%) DN+|MTP- transcripts partially mapped to the reference genome (20% < query coverage < 95%).

Finally, we found 144 (3%) DN+|MTP- transcripts that can be completely mapped to a novel sequence identified by the MTP approach. These are likely the result of the more limited genomic context in which annotation is performed in the MTP approach.

Together these results suggest that the higher number of nonreference genes detected by the DN approach stems from: (1) its ability to predict genes in reference sequences; and (2) its ability to analyze partially novel genomic sequences.

## Supplementary note 3 – analysis of DN-|MTP+ nonreference pan-genes in *A. thaliana*

All genomic sequences annotated in the MTP approach are extracted from genome assemblies, and as such are necessarily also annotated by the DN approach. Therefore, the existence of 574 DN-|MTP+ nonreference genes might be surprising at first, and points to methodological biases in one or both construction approaches (Supplementary Figure S1). To investigate these methodological biases, we first mapped protein sequences of DN-|MTP+ pan-genes to those of DN+|MTP- pan-genes. 60 proteins (10%) show high similarity to DN+|MTP- proteins (percent identity > 95%), however most are in fact truncated versions of those DN+|MTP- genes. Such cases likely occur when novel genomic regions flanked by reference-homologous regions are annotated by the DN procedure.

We performed another analysis, this time mapping DN-|MTP+ protein sequences to protein sequences derived directly from whole genome annotations of the DN pan-genome, before orthology clustering and selection of representative genes (see Figure 1). 381 of the DN-|MTP+ (66%) show very high similarity (percent identity > 95% and length difference < 10%) to annotated DN proteins. This indicates that the corresponding nonreference genes were in fact detected by the DN approach, however they were not selected as the representative of the orthology cluster to which they belong, for example because this cluster contains a reference gene. The remaining 24% may result from over-annotation by the MTP approach.

## Supplementary note 4 – analysis of gene presence-absence discrepancies between *A. thaliana* DN and MTP pan-genomes

Due to the high number of genes that were classified as present in certain accessions in the MTP pan-genome but absent in the DN pan-genome, we further investigated the cause of these discrepancies. These inconsistencies occur when the reads from a given ecotype reliably cover the genomic region containing an annotated gene, and hence the gene is called present by the MTP pipeline. In contrast, that gene is not detected by the DN pipeline when the genome assembly of the same ecotype is annotated. The absence of this gene may stem from: (1) the absence of the gene sequence in the genome assembly of the relevant ecotype, or (2) the absence of a gene model in the genome annotation of that ecotype. The latter may result from a mutation in this region causing it not to be annotated as a coding gene, or from insufficient evidence for detecting the gene during the annotation process. To determine which of these scenarios is more likely, for each discrepancy we searched for the relevant transcript (cDNA) sequence, obtained from the MTP pan-genome, in the genome assembly of the respective ecotype. 51.7% of the transcripts could be reliably mapped (query coverage > 90%), indicating the presence of the genomic sequence, but absence of an annotated gene in the DN pan-genome. Transcripts were also searched against high-quality assemblies of the same ecotypes (see Methods section). An additional 20.2% could be mapped to the high-quality assemblies, but not to the 50× ones, indicating discrepancies resulting from incomplete genome assemblies. The remaining 28.1% may result from erroneous presence calls by the MTP approach, probably due to lenient read mapping cutoffs (Supplementary note 5 and Supplementary Figure S3). Alternatively, they may be caused by issues related to orthology clustering (Supplementary Note 6 and Supplementary Figure S5).

## Supplementary note 5 – analysis of gene presence-absence detection thresholds in the Map-to-pan approach

The last step of the MTP pan-genome construction procedure (Figure 1) is aimed at determining the presence of all reference and nonreference pan-genes in each of the examined accessions to create the gene PAV matrix. This is achieved through mapping of short sequencing reads to the genomic reference and nonreference pan-genome sequences and analysis of coverage patterns for each gene. This usually requires the choice of values for two parameters:

1. The depth threshold – the minimal number of mapped reads required to label a given position in the gene sequence "covered".
2. The coverage threshold – the minimal percentage of the gene sequence labeled as "covered" required to assign the gene as present in a given accession.

Different pan-genome studies have arbitrarily used different thresholds, without reporting the effects of the value choice on the obtained pan-genome. We therefore analyzed the effect of these two parameters to gain a general understanding on how they impact gene presence-absence detection by the MTP approach, and the inferred pan-genome composition.

We began by examining the effect of the depth threshold parameter on the percentage of gene coverage across all pan-genes of a single *A. thaliana* ecotype (An-1), using 50× sequencing data. As expected, using depth thresholds of 1, 5, 10, 15, 20, 30, 40, and 50 reads resulted in considerably different distributions of gene coverage, with lower threshold values leading to high gene coverage values (Supplementary Figure S3A). Notably, intermediate depth thresholds (20,30,40) resulted in higher variation among genes compared to more extreme values (1-15 and 50). Next, we computed the percentage of genes determined as present in the examined ecotype out of the total 27,960 pan-genes, using different values of the depth and coverage thresholds (Supplementary Figure S3B). Using lenient depth thresholds between 1 and 15 resulted in gene presence percentage > 90% for any coverage threshold < 95%. Using a depth threshold of 20 and coverage threshold of 75% resulted in a gene presence percentage similar to the one observed using the DN approach (87%). Depth thresholds of 30 or higher resulted in unrealistically low gene presence percentages for almost any coverage threshold.

We further examined the effect of the threshold parameters on two measures related to the pan-genome composition (using the 50× pan-genome): the percentage of core pan-genes (present in all eight accessions; Supplementary Figure S3C) and the overall gene occupancy (see the main text for details; Supplementary Figure S3D). The observed patterns were similar to those described above, with low depth and coverage threshold values resulting in high percentage of core genes and gene occupancies. The percentage of core genes detected by the DN approach (73%) was observed with depth and coverage thresholds considerably more stringent than commonly used (e.g. depth threshold 15 and coverage threshold 55%, or depth threshold 10 and coverage threshold 80%).

In most studies which applied the MTP approach (including this one), the depth threshold was specified as an absolute number of reads. However, the choice of this parameter value should obviously be based on the amount of available sequencing data, and thus may be specified as a fraction of the mean sequencing depth. We therefore analyzed the effect of the mean sequencing depth when using various depth fraction thresholds. For the purpose of this analysis, we fixed the coverage threshold at 50% and computed the mean sequencing depth against the percentage of gene presence in a single ecotype (An-1), using different depth fraction thresholds (0.1, 0.3, 0.5, 0.7, 0.9; Supplementary Figure S3E). The sequencing depth appears to have only a modest effect on the gene presence-absence inferences, indicating that setting the threshold as a fraction of the mean depth rather than as an absolute number is an effective way to perform this type of analysis.

## Supplementary note 6 – *A. thaliana* genome assembly and contamination analysis

During the analysis of genome assemblies, we noticed that the assembly of the ecotype Ler shows considerably lower N50 values and larger assembly size compared to other ecotypes in most-data sets (Figure 3A and Supplementary Table S4). We suspected that this may be the result of biological sequencing contamination, specifically with aphid DNA. To test this, we generated a data base of whole genome sequences of five Hemiptera species (*Cimex lectularius, Rhodnius prolixus, Bemisia tabaci, Acyrthosiphon pisum,* and *Trialeurodes vaporariorum*), obtained from ENSEMBL Metazoa Release 52. All Ler contigs from the 50× data set which could not be reliably mapped to the *A. thaliana* reference genome during Panoramic's reference-guided assembly step were searched for within the Hemiptera DB, using Blastn. As expected, we found that Ler contigs with high similarity to Hemiptera genomes (% identity > 70) accounted for 52,762,315 bp out of the total 60,862,882 nonreference sequences (87%), indicating that the majority of nonreference genome sequences derives from aphid DNA sequencing contamination, assembled into contigs.

We tested the effect of this contamination on gene prediction results by mapping the transcript sequences of nonreference genes from the DN 50× pan-genome to the Hemiptera DB, using Minimap2. None of the transcripts showed significant similarity to Hemiptera genomes, indicating no gene models of aphid origin were incorporated into the pan-genome.

## Supplementary note 7 – analysis of the effect of read-mapping software in the Map-to-pan and iterative mapping approaches

Read mapping occurs at two steps in the IA pipeline:

1. Reads of each sample are iteratively mapped to the reference genome to detect nonreference (unmapped) reads, which are subsequently assembled into nonreference contigs.
2. Once a pan-genome sequence had been obtained and annotated, reads from each sample are mapped to the pan-genome to determine the presence and absence of pan-genes in the input accessions, thus constructing the final gene PAV matrix.

In the MTP approach, only step 2 is required.

While many read-mapping algorithms exist, the two most commonly used in the context of pan-genome construction are BWA MEM and Bowtie2. We therefore examined the effect of the choice of read-mapping algorithm at these two steps by constructing *A. thaliana* pan-genomes from the same 50x sequencing data, using either of the software tools and comparing them to one another.

**Step 1**: We found that Bowtie2 was considerably more restrictive in mapping reads to the reference, which resulted in a higher fraction of reads detected as nonreference (unmapped; Supplementary Table S10). The number of nonreference reads per accession increased by 30% on average when using Bowtie2, compared to BWA. Interestingly, this resulted in an average two-fold increase in the total length of assembled nonreference contigs per accession. Nonreference contigs generated based on the Bowtie2 mapping were 10% longer on average than those based on BWA mapping. The total length of the nonredundant nonreference genomic sequences was 26% larger in the Bowtie2 pan-genome (100.45 Mb) compared to the BWA pan-genome (79.54 Mb) and they were, on average, 8% longer. Annotation of these nonreference sequences resulted in 959 nonreference pan-genes in the Bowtie2 pan-genome, compared to only 187 in the BWA pan-genome. We therefore conclude that the choice of read-mapping algorithm has a major effect when using the IA approach.

**Step 2:** We assessed the effect of the read-mapping algorithm on gene presence-absence detection by performing the same set of steps on the same sequencing data (50x data of the An-1 accession) and pan-genome, using either BWA or Bowtie2. Reads were first mapped to the pan-genome (including both the reference genome and the nonreference contigs). Results were highly similar, with BWA mapping only 1% more reads than Bowtie2. We therefore suggest that the difference between BWA and Bowtie2 is mainly observed when mapping highly diverged sequences, as done in step 1. Here, however, most mappings are of very high quality and thus the agreement between the algorithms is high. Gene presence was detected based on read mappings (depth threshold: 3; coverage threshold: 0.4; see Supplementary Note 5 for details). Out of 28,254 pan-genes, discrepancies were found in 126 (0.4%), out of which 116 were detected as present based on Bowtie2 but absent based on BWA, and 10 were detected as absent based on Bowtie2 but present based on BWA. In conclusion, we found that the choice of read mapping algorithm has a relatively modest effect on the gene presence-absence detection step, compared to other factors and parameters.

## Supplementary note 8 – analysis of the MCL inflation parameter in the De novo approach

The size and composition of pan-genomes constructed using the DN approach, as well as the gene presence-absence inference in specific accessions, are highly dependent on the orthology clustering step (see main Figure 1). In this step, predicted protein sequences derived from genome annotations of all accessions are clustered into orthology groups (orthogroups) based on sequence similarity. Each orthogroup represents a pan-gene, and the pan-genome presence-absence matrix is created based on the existence of representative genes from each accession in the pan-gene clusters. Orthology clustering is an important task in comparative genomics and multiple methods and software tools have been developed to address it. One common technique is based on the Markov clustering (MCL) algorithm, with tools such as OrthoMCL and OrthoFinder applying it as part of their workflows. The main parameter controlling the behavior of MCL is called the "inflation" parameter. When values of this parameter are increased, the "tightness" or "granularity" of the clustering process is enhanced, practically resulting in a higher number of smaller clusters. Most previous pan-genome studies which applied the MCL algorithm simply used the default values set by OrthoMCL and OrthoFinder (1.5), therefore the optimal value and the exact effect of this parameter are still unknown.

We examined the effect of the inflation parameter on the resulting pan-genome by repeating the final steps of the DN procedure on the same data set (*A. thaliana* 50×) using a range of values for the inflation parameter: 1.0, 1.2, 1.4, … 4.8, 5.0. We chose this set of values since they are in the recommended range found in the MCL manual (<http://micans.org/mcl/man/mcl.html>). For each inflation value, we report the number of nonreference pan-genes and the percentage of core, shell, and singleton pan-genes. As can be observed in Supplementary Figure S5, both the size and composition of the pan-genome are affected by the choice of the inflation parameter value. As expected, increasing the value resulted in a larger number of smaller orthogroups. Since the number of reference genes is constant, this leads to more clusters not containing a reference gene and thus to higher numbers of nonreference pan-genes (and larger pan-genomes). The number of nonreference pan-genes was 4.8 times larger when setting the inflation value to 5 (15,853) then when setting it to 1 (3,322). Similarly, as higher inflation values tend to break orthogroups, more pan-genes are inferred to be absent in some of the accessions and hence the effect on pan-genome composition. Specifically, the proportion of core pan-genes decreased as the inflation value increased, ranging between 78% and 50%. Interestingly, the proportion of shell pan-genes remained nearly constant (17-23%) whereas the proportion of singletons increased (5-32%). This indicates that higher inflation values tend to remove only one gene from orthogroups, resulting in singletons. Importantly, within inflation value range 1-2, only a moderate effect on pan-genome size and composition was observed (3,322-5,207 nonreference pan-genes and 70-78% core pan-genes), while larger values lead to considerably different results. As most comparative studies use inflation values within the range of 1-2, this parameter is unlikely to have caused significant bias in previous publications.
